# Golden berry 4β-hydroxywithanolide E prevents tumor necrosis factor α-induced procoagulant activity with enhanced cytotoxicity against human lung cancer cells

**DOI:** 10.1038/s41598-021-84207-8

**Published:** 2021-02-25

**Authors:** Kan-Yen Hsieh, Ju-Ying Tsai, Ya-Han Lin, Fang-Rong Chang, Hui-Chun Wang, Chin-Chung Wu

**Affiliations:** 1 Graduate Institute of Natural Products, Drug Development and Value Creation Research Center, Kaohsiung Medical University, Kaohsiung, Taiwan; 2 Department of Medical Research, Kaohsiung Medical University Hospital, Kaohsiung, Taiwan

**Keywords:** Pharmacodynamics, Tumour-necrosis factors, Inflammation, Coagulation system

## Abstract

Inflammation in the tumor microenvironment is positively correlated with cancer progression and metastasis as well as the risk of thromboembolism in lung cancer patients. Here we show, in human non-small cell lung cancer (NSCLC) cell lines, the master inflammatory cytokine tumor necrosis factor (TNF-α) induced tissue factor expression and procoagulant activity, and these effects were potently inhibited by 4β-hydroxywithanolide E (4HW), a natural compound isolated from *Physalis peruviana*. Furthermore, combination of 4HW and TNF-α caused synergistic cytotoxicity against NSCLC cells by inducing caspase-dependent apoptosis. The underlying mechanism by which 4HW reverses the procoagulant effect of TNF-α but enhances its cytotoxic effect appears to be due to inhibition of NF-κB, which is a key switch for both inflammation-induced coagulation and cell survival. Our results suggest that 4HW may have a potential application for treating inflammation-derived cancer progression and cancer-associated hypercoagulable state.

## Introduction

Growing evidence supports a link between inflammation and cancer^1^. The inflammatory cells and cytokines found in the tumor microenvironment can enhance tumor growth as well as progression, and suppress anti-tumor responses^2^. Tumor necrosis factor-α (TNF-α) is a major inflammatory cytokine that is produced by tumor cells and/or tumor-associated immune cells such as activated macrophages and lymphocytes^3^. TNF-α was first identified as a potential anti-tumor agent, as it can induce and trigger apoptosis of cancer cells in high doses; however, due to significant toxicities and lack of efficacy, the clinical trials of systemic administration of TNF-α for treating cancer have been disappointing^4^. Recent studies have even suggested that constitutive expression of TNF-α within the inflammatory tumor microenvironment plays a pro-tumorigenic role by enhancing cancer cell survival, angiogenesis, and metastasis formation^5–7^. In patients with non-small cell lung cancer (NSCLC), high mRNA expression of TNF-α was found in pleural effusion and tumor tissue^8^. Additionally, high plasma levels of this cytokine have been associated with poor prognosis in lung cancer patients^9,10^.

Hypercoagulopathic syndromes such as venous thromboembolism are the second leading cause of death in cancer patients^11^. Lung cancer has among the highest morbidity and mortality associated with venous thromboembolism of all cancer types^12,13^. This risk is even more in lung cancer patients who receive surgery or chemotherapy^14,15^. The poor prognosis of lung cancer patients complicated with thromboembolism was positively correlated with inflammation markers^16,17^, suggesting that inflammation is a contributing factor to both hypercoagulability and cancer progression. TNF-α is known as a potent inducer of tissue factor (TF), which is the primary cellular initiator of blood coagulation, in tumor cells and tumor-associated macrophages^18^. Moreover, TF of tumor origin accounts for cancer procoagulant activity and is considered to be the trigger of venous thromboembolism in cancer patients^19,20^. Current anticoagulants, such as low molecular weight heparins, are effective in reducing cancer-associated thrombosis; however, these agents suffer from significant limitations, including bleeding complications and reoccurrence of thrombosis^21,22^. These limitations have encouraged the search for new antithrombotic strategies based on understanding of the molecular mechanisms in the interactions between cancer-associated inflammation and coagulation^23^.

4β-hydroxywithanolide E (4HW) is a natural compound isolated from an edible plant, *Physalis peruviana* (golden berry). Previous studies have shown that 4HW has cytotoxic effect against human lung, breast, oral, pancreatic, prostate and renal cancer lines in vitro^24–28^. 4HW also inhibited tumor growth in mouse xenograft models of liver and colorectal cancer^29,30^. Besides anticancer effects, low-dose 4HW exhibits potent anti-inflammatory activity in vitro and in vivo through inhibiting NF-κB or Akt signaling^31–33^. In this study, we investigated the inhibitory effect of 4HW on the hypercoagulable states of NSCLC cells under inflammatory conditions. Our results indicate that 4HW is able to prevent TNF-α-induced TF expression and procoagulant activity in NSCLC cells. Moreover, a synergistic cytotoxic effect of 4HW and TNF-α against NSCLC cells was demonstrated.

## Results

### 4HW inhibits TNF-α-induced TF expression and activity in NSCLC cells

**Figure 1:**
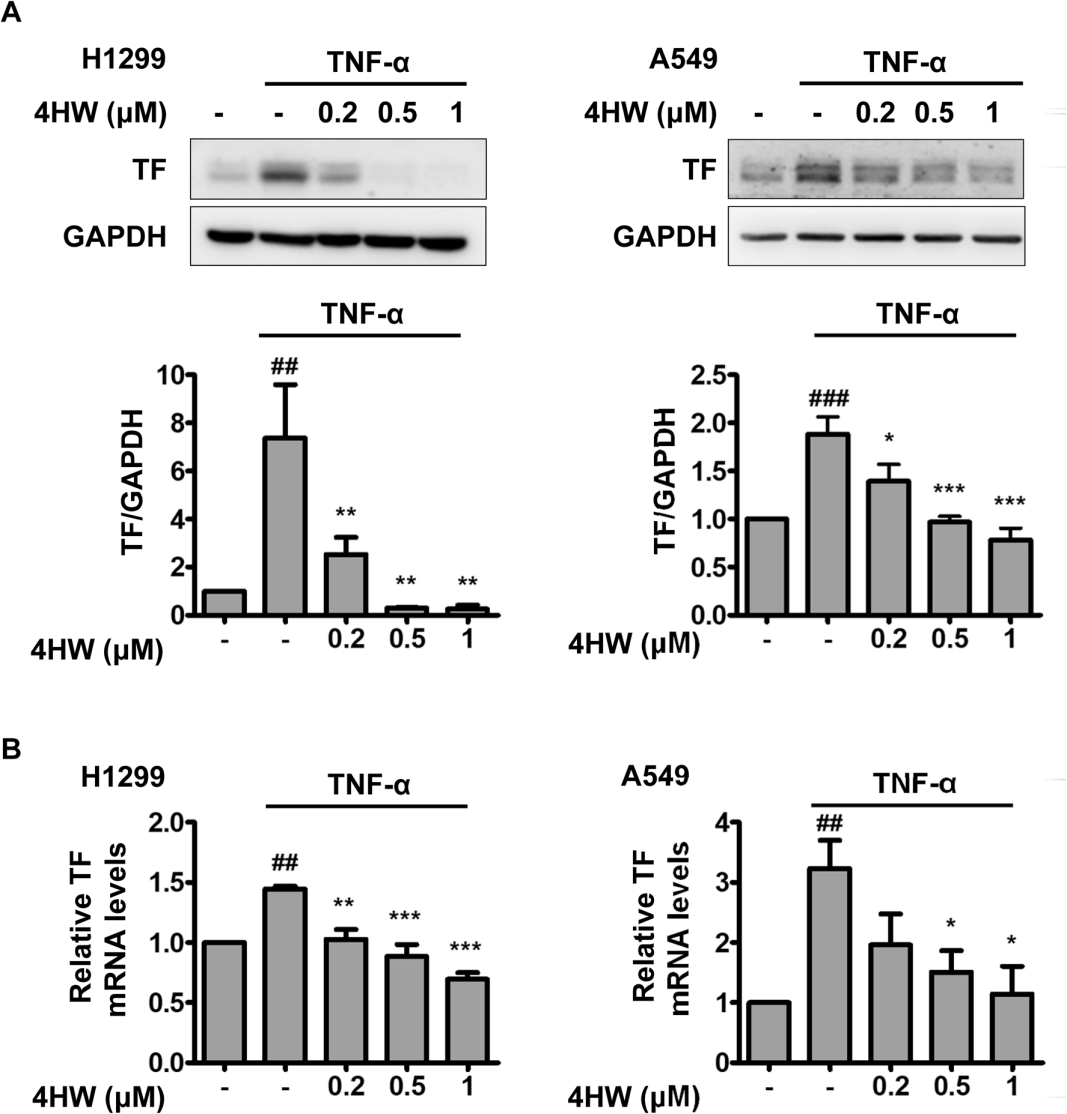
4HW inhibits TNF-α-induced TF protein and mRNA expression in NSCLC cells. H1299 and A549 cancer cells were pretreated with DMSO (vehicle) or 4HW for 1 h, followed by incubation with or without TNF-α 20 ng/mL for 12 (**A**) or 2 h (**B**). The protein and mRNA levels of TF were evaluated by immunoblotting and real-time RT-PCR, respectively. Full-length blots are shown in the Supplementary information S1. All results are presented as mean ± SEM (n ≥ 3). *P < 0.05, **P < 0.01, ***P < 0.001 as compared with the TNF-α control group. ^##^*P* < 0.01, ^###^*P* < 0.001 as compared with the untreated control group.

As shown in Fig. 1A, the NSCLC H1299 and A549 cells constitutively expressed TF in untreated conditions. Stimulation of the NSCLC cells with TNF-α (20 ng/mL) for 12 h resulted in a further increase in the protein levels of TF. Pretreatment of 4HW (0.2–1 μM) potently inhibited TNF-α-induced TF protein expression in a concentration-dependent manner. Next, TF mRNA expression in the NSCLC cells was determined by using real time RT-PCR (Fig. 1B). 4HW prevented TNF-α-induced TF mRNA in the same concentration range as that for inhibiting TF protein expression, suggesting that 4HW inhibition of TF expression is mediated at the transcriptional level.

**Figure 2:**
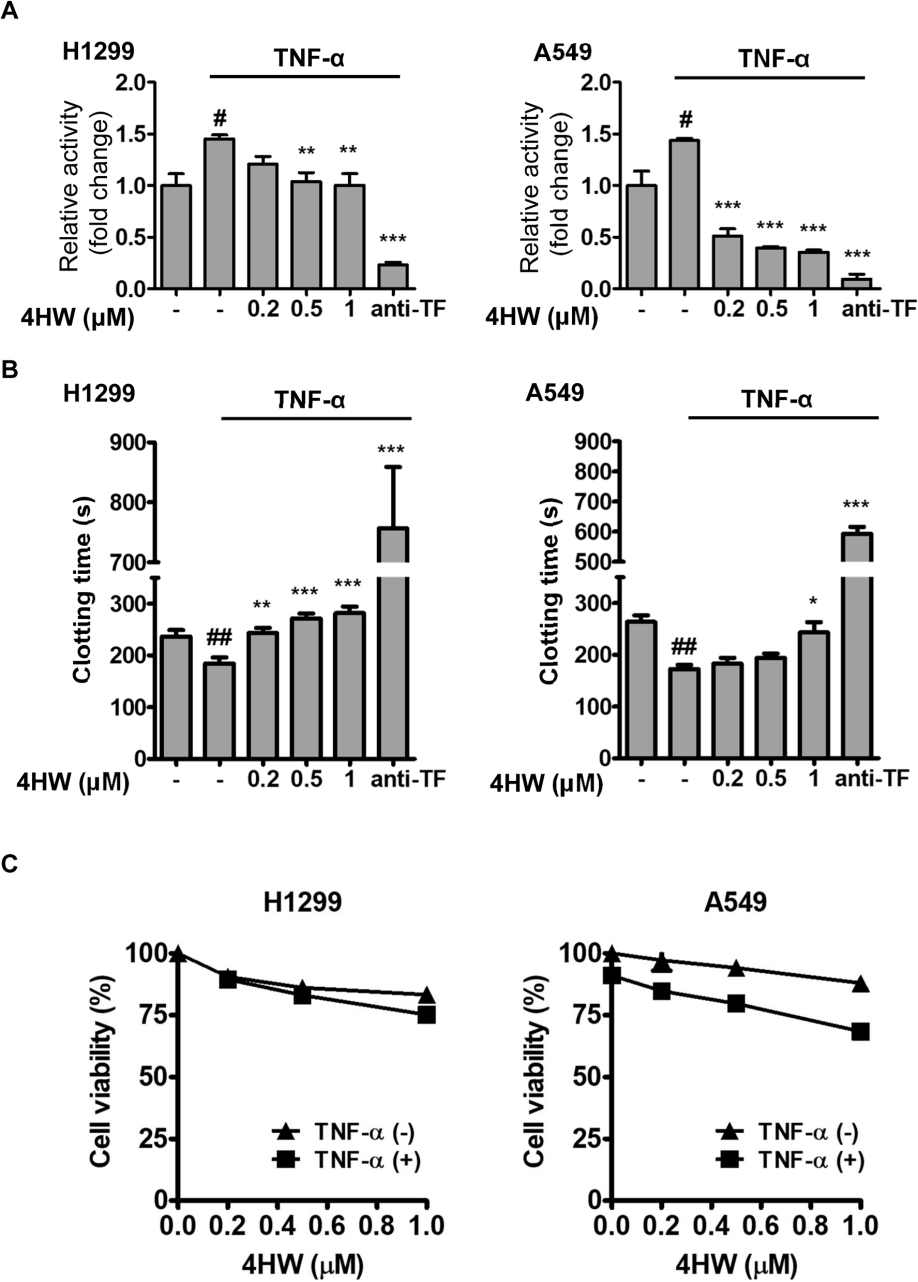
4HW inhibits TNF-α-induced procoagulant activity in NSCLC cells. H1299 and A549 cancer cells were pretreated with 4HW or DMSO (vehicle control) for 1 h, followed by incubation with or without TNF-α 20 ng/mL for 24 h. The treated cells were subjected to amidolytic assay (**A**), plasma clotting assay (**B**), and MTT assay (**C**) as described in “Methods”. The relative amidolytic activity is presented as a fold change relative to untreated control cells. Anti-TF antibody (20 μg/mL) was used as a positive control. Results are presented as mean ± SEM (n ≥ 3). **P < 0.01, ***P < 0.001 as compared with the TNF-α control group. ^#^*P* < 0.05, ^##^*P* < 0.01, ^###^*P* < 0.001 as compared with the untreated control group.

In order to examine if the inhibition of TF expression by 4HW is accompanied by concomitant loss of biological function, the TF-dependent procoagulant activity of H1299 and A549 cells were assessed by amidolytic assay and plasma clotting assay. In the amidolytic assay, the TF activity was measured by incubating cancer cells with purified coagulation factors VIIa and X. In this system, TF on the surface of cancer cells forms a complex with factor VIIa that activates factor X and leads to the lysis of an artificial substrate of Xa. Figure 2A shows that TNF-α (20 ng/mL) caused an increase in the TF activity of both cell lines, and the increased TF activity was concentration-dependently inhibited by 4HW treatment. In the clotting assay, recalcified human plasma was incubated with cancer cells in the absence or presence of TNF-α. Stimulation of the NSCLC cells with TNF-α led to a significant decrease in the clotting times compared with untreated groups (Fig. 2B). 4HW treatment reversed TNF-α’s effect on the clotting times. Of note, at the concentrations (0.2–1 μM) and time (24 h) used, 4HW alone or in the presence of TNF-α reduced the cell viability by up to 16.9% in H1299 cells and 31.5% in A549 cells, suggesting that the inhibitory effect of 4HW on TF-mediated procoagulant activity was not mainly due to its cytotoxicity toward cancer cells (Fig. 2C).

### 4HW enhances TNF-α-mediated cytotoxicity in NSCLC cells

**Figure 3:**
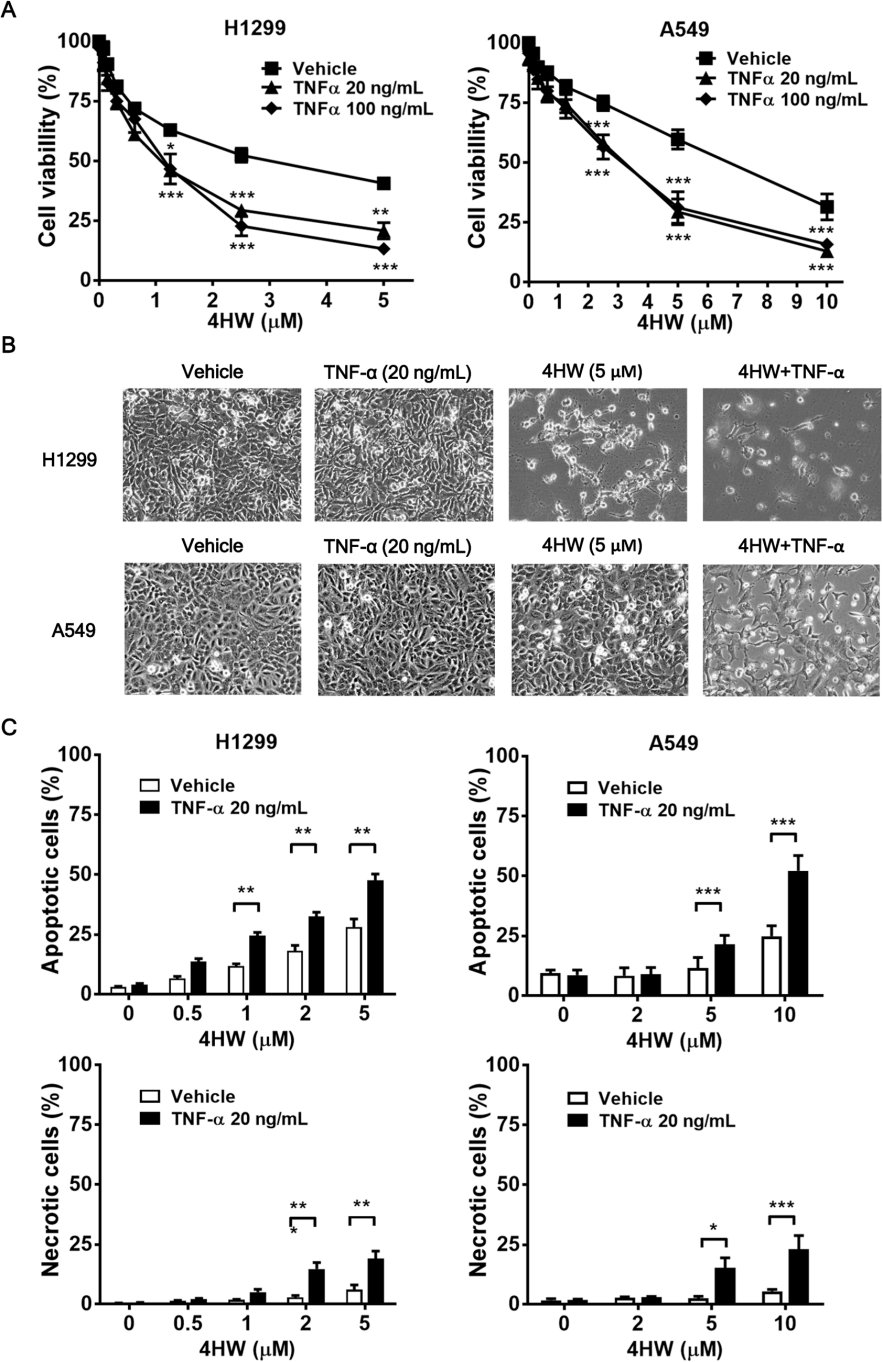
Combination of 4HW with TNF-α causes enhanced cytotoxicity against NSCLC cells. H1299 and A549 cancer cells were treated with 4HW alone or in combination of TNF-α for 48 h, and DMSO was used as a vehicle control. The cytotoxicity was evaluated by MTT assay (**A**), morphological changes (**B**), and annexin V/PI double staining assay (**C**), respectively. Apoptotic cells were indicated as annexin V-positive/PI-negative or -positive, while necrotic cells were indicated as annexin V-negative/PI-positive. Representative flow cytometry dot plots are shown in Supplementary Figure S1. Results are presented as mean ± SEM (n ≥ 3). *P < 0.05, ***P < 0.001 compared to the DMSO (vehicle) control group.

Many types of cancer cells, including NSCLC cells, are relatively resistant to TNF-α-mediated cytotoxicity^34^. MTT assay revealed that, in both H1299 and A549 cells, TNF-α at the concentrations up to 100 ng/mL did not cause significant cytotoxicity at 48 h of treatment (Fig. 3A). In contrast, 4HW treatment decreased the cell viability with the IC_50_ value of 3.62 ± 0.15 μM for H1299 cells and 6.06 ± 0.08 μM for A549 cells respectively. Furthermore, combination of 4HW and TNF-α (20 ng/mL) caused synergistic cytotoxicity in both NSCLC cell lines, although larger doses of 4HW and a longer period of treatment than those used in TF experiments were required to achieve this effect (Fig. 3A). Morphological changes such as cell rounding or shrinking were visible in NSCLC cells treated with 4HW, and more evident in those treated with 4HW plus TNF-α (Fig. 3B). The cell death was further investigated using annexin V/PI double staining. Figure 3C and Supplementary Fig. S1 show that 4HW alone induced apoptosis and, to a lesser extent, necrosis in both H1299 and A549 cell lines. The effect of 4HW to induce apoptotic and necrotic death was further enhanced when in combination with TNF-α.

### 4HW-enhanced cytotoxicity of TNF-α is mainly via caspase-dependent apoptosis but not necroptosis

**Figure 4:**
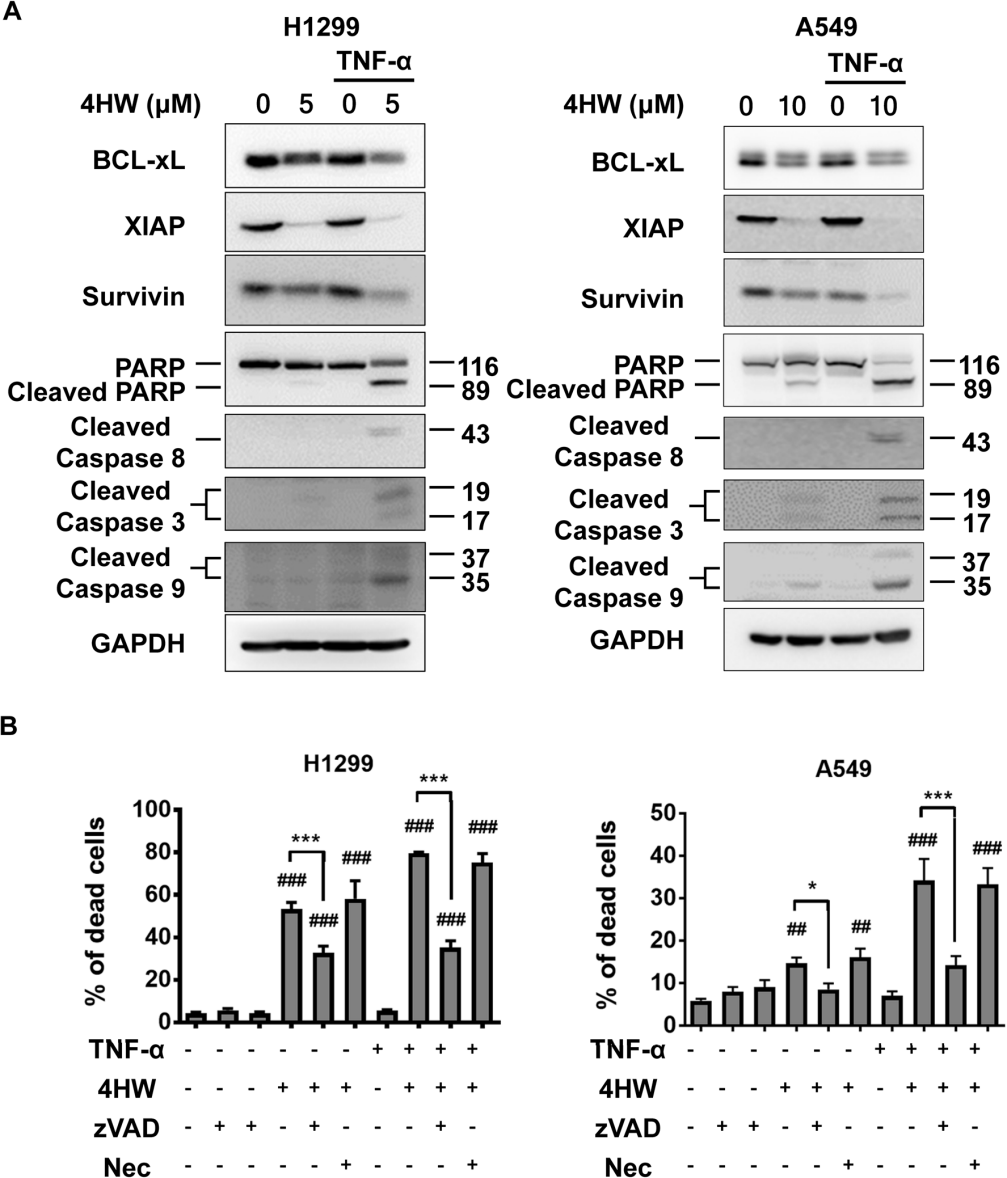
Cell death caused by 4-HW and TNF-α is mainly due to caspase-dependent apoptosis. (**A**) NSCLC cells were treated with 4HW or DMSO (vehicle control) in the presence or absence of TNF-α (20 ng/mL) for 24 h, the apoptosis-related biomarkers were then determined by immunoblotting. Full-length blots are presented in the Supplementary information S1. (**B**) NSCLC cells were pretreated with the pan-caspase inhibitor Z-VAD(OMe)-FMK (Z-VAD, 25 μM) or the necroptosis inhibitor necrostatin-1 (Nec, 20 μM), and treated with 4HW and/or TNF-α for 48 h. The apoptotic cells were evaluated by annexin V/PI double staining. Representative flow cytometry dot plots are shown in Supplementary Figure S2. Results are presented as mean ± SEM (n ≥ 3). *P < 0.05, ***P < 0.001 as compared with the indicated group. ^##^*P* < 0.01, ^###^*P* < 0.001 as compared with the untreated control group.

To elucidate the mechanism of enhanced cytotoxicity by 4HW in combination with TNF-α, several protein molecules involved in the process of cell survival and apoptosis were determined using immunoblotting. Bcl-xL, survivin, and XIAP are anti-apoptotic proteins upregulated in a variety of human cancers and contributing to resistance to TNF-α^35,36^. 4HW alone, and to a larger extent in combination with TNF-α (20 ng/mL), reduced the levels of anti-apoptotic proteins in both NSCLC cell lines (Fig. 4A). Consistent with decreased anti-apoptotic proteins, the combined treatment of 4HW with TNF-α led to stronger activation of caspase-8, -9, and -3 as well as cleavage of PARP than 4HW alone. The activation of caspase-8 and -9 suggests that both extrinsic and intrinsic apoptosis pathway were involved. Since the TNF-α–TNF receptor signaling pathway can trigger either apoptosis or necroptosis^37^, we used the pan-caspase inhibitor Z-VAD(OMe)-FMK and the necroptosis inhibitor necrostatin-1 to examine if 4HW/TNF-α-induced cell death is dependent on apoptosis and/or necroptosis. As shown in Fig. 4B and Supplementary Fig. S2, the cell death caused by 4HW alone or in combination with TNF-α was largely prevented by Z-VAD(OMe)-FMK. In contrast, necrostatin-1 was ineffective. These results suggest that 4HW/TNF-α-induced cell death is mainly due to caspase-dependent apoptosis.

### Inhibition of NF-κB contributes to 4-HW’s effects on TNF-α-induced procoagulant activity and apoptosis

**Figure 5:**
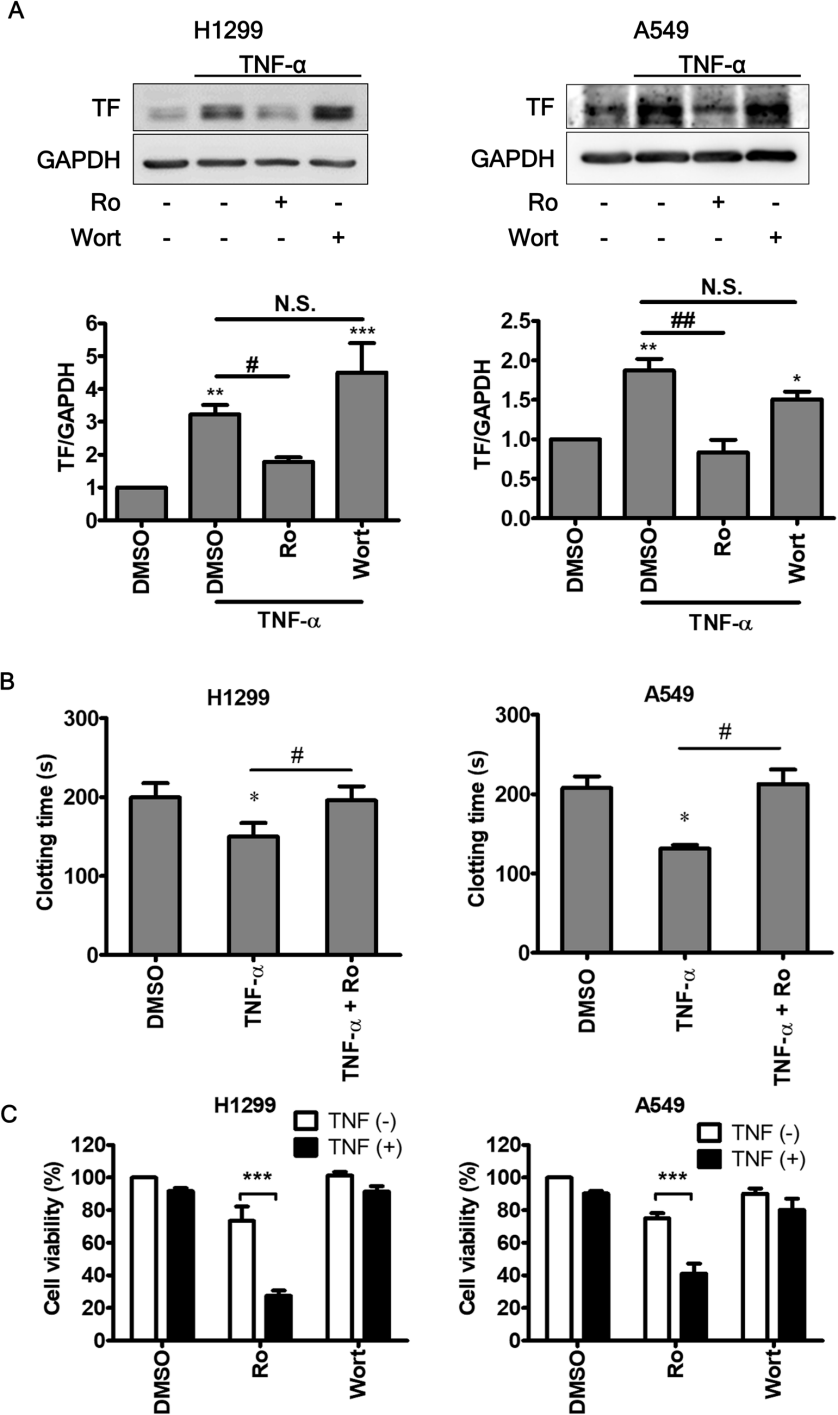
Inhibition of NF-κB reduces TNF-α-induced procoagulant activity and enhances cell death. NSCLC cells were pretreated Ro 106-9920 (10 μM), wortmannin (0.1 μM), or DMSO (vehicle control) for 1 h and then treated with TNF-α (20 ng/mL) for 12 (**A**), 24 (**B**), and 48 (**C**) hours, respectively. (**A**) The protein expression of TF was determined by immunoblotting. Full-length blots are presented in the Supplementary information S1. (**B**) Cancer cell-associated procoagulant activity was determined by plasma clotting assay. (**C**) Cell viability was assessed by MTT assay. Results are presented as mean ± SEM (n ≥ 3). *or ^#^*P* < 0.05, **or ^##^*P* < 0.01, ***P < 0.001.

In NSCLC cells, NF-κB is the major downstream effector of TNF-α^38^. In order to confirm if NF-κB activation contributes to TNF-α-induced TF expression and affects cell fate in NSCLC, a specific inhibitor of NF-κB, Ro 106-9920, was used. Pretreatment of H1299 or A549 cells with Ro 106-9920 prevented TNF-α-induced TF expression (Fig. 5A). Consistently, the shortened clotting time caused by TNF-α was also completely reversed in Ro 106-9920-treated NSCLC cells (Fig. 5B). Furthermore, Ro 106-9920 in combination with TNF-α led to an enhanced cell killing effect (Fig. 5C). In contrast to NF-κB, inhibition of the PI3K/Akt pathway by wortmannin had no effect on TNF-α. Because the effect of Ro 106-9920 on TNF-α-induced procoagulant activity and cytotoxicity was similar to that of 4HW, this suggests that NF-κB is a potential target of the latter.

**Figure 6:**
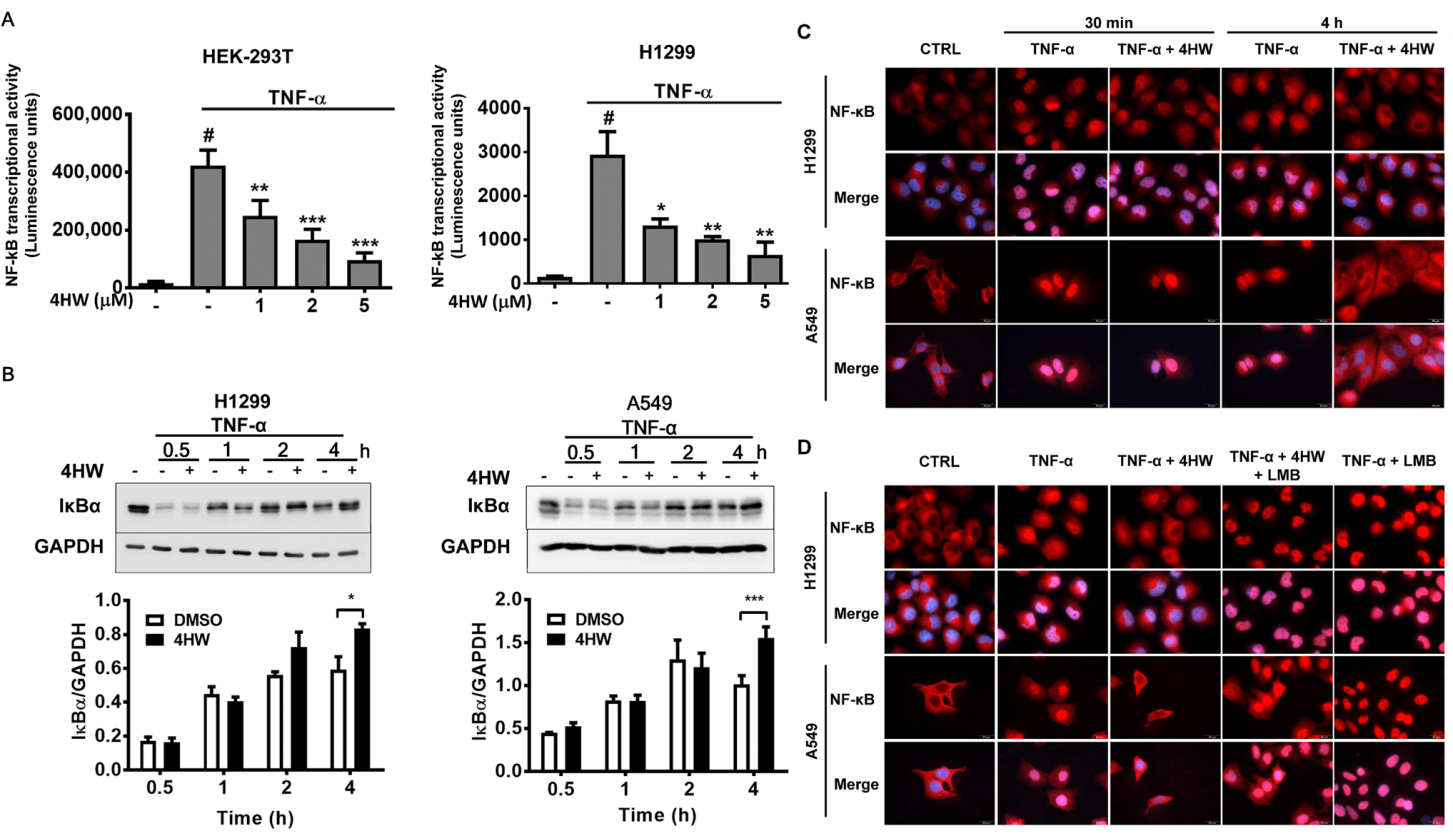
The effect of 4HW on TNF-α-induced NF-κB signaling. (**A**) HEK 293T or H1299 cells transfected with a NF-κB luciferase reporter were pretreated with 4HW or DMSO (vehicle control) and then stimulated with TNF-α (20 ng/mL) for 6 h. NF-κB-dependent luciferase activity was determined as described in “Methods”. (**B**) NSCLC cells treated with 4HW (1 μM for H1299, 2 μM for A549) or DMSO (vehicle control) were stimulated with TNF-α for indicated periods, then protein expression of IκB was determined by immunoblot blotting. Full-length blots are shown in the Supplementary information S1. All results are presented as mean ± SEM (n ≥ 3). *or ^#^*P* < 0.05, **P < 0.01, ***P < 0.001. (**C**,**D**) NSCLC cells were treated as (**B**), and nuclear translocation of NF-κB p65 (red) was determined by immunofluorescence microscopy. Nuclei were counterstained with DAPI (blue). The images were overlapped (merge) to determine translocation. *LMB* leptomycin B.

The effect of 4HW on NF-κB activation was firstly examined in a luciferase reporter cell model. Figure 6A shows that TNF-α markedly induced luciferase activity in HEK 293 T cells transfected with an NF-κB-luciferase reporter gene, and the induced activity was prevented by 4HW in a concentration-dependent manner. A similar effect of 4HW was also observed in NF-κB-luciferase reporter-transfected H1299 cells. Next, 4HW’s action on NF-κB signaling was further investigated in NSCLC cells. As shown in Fig. 6B, stimulation of both A549 and H1299 cells with TNF-α led to a rapid degradation of IκB within 30 min, followed by reappearance of this protein after 1–4 h. Surprisingly, pretreatment of 4HW had no effect on the degradation of IκB in response to TNF-α. 4HW also failed to affect significantly the reappearance of IκB for up to 2 h, but did cause an increase in IκB levels after 4 h of TNF-α stimulation. We subsequently investigated the nuclear translocation of NF-κB p65 by using immunofluorescence microscopy. Upon TNF-α stimulation, p65 rapidly translocated from the cytoplasm into the nucleus within 30 min, followed by a slow and gradual decline of nuclear p65 over 4 h. Pretreatment of 4HW did not affect nuclear translocation of p65 within 30 min, but enhanced the subsequent decline of p65 levels in the nucleus after 4 h of TNF-α stimulation (Fig. 6C). Because 4HW did not affect TNF-α-induced nuclear translocation of p65, this suggests that it might act at the level of nuclear export rather than import. To examine this hypothesis, exportin 1 (XPO1, also known as CRM1), a mediator of protein export from the nucleus to the cytoplasm, was inhibited by either pharmacological or genetic approaches. As shown in Fig. 6D, the inhibitory effect of 4HW on nuclear p65 accumulation was blunted by the XPO1 inhibitor leptomycin B (LMB). Additionally, knockdown of XPO1 with short hairpin RNA (shRNA) in the NSCLC cells had the same effect as that produced by LMB (Supplementary Fig. S3). Taken together, these results indicate that 4HW inhibits TNF-α-induced NF-κB activation by reducing NF-kB nuclear retention.

## Discussion

Cancer-associated inflammation not only contributes to tumor progression and metastasis, but also to the hypercoagulable state in patients with NSCLC^39–41^, so interfering with the interactions between cancer-associated inflammation and coagulation could represent a novel strategy for treating NSCLC and preventing venous thromboembolism. In the present study, two NSCLC cell lines, H1299 and A549 cells, were examined because they bear a p53 mutation and a KRAS mutation, respectively. Mutations in p53 tumor suppressor gene and KRAS oncogene are common in NSCLC^42^, and are responsible for chemoresistance and tumorigenesis^43^. Additionally, TF expression is regulated positively by mutant KRAS and negatively by p53^42,44^. Here, we show that the expression and activity of TF in either p53-deficient (H1299) or KRAS-mutant (A549) NSCLC cells can be further enhanced in the presence of TNF-α. Moreover, we have demonstrated that 4HW, a natural compound with anti-inflammatory and anticancer effects, significantly prevented TNF-α’s procoagulant effect at sub-micromolar concentrations by reducing TF mRNA and protein expression in NSCLC cells. These findings support a role for TNF-α in lung cancer hypercoagulability and suggest inhibition of TNF-α-induced TF as a potential therapy for cancer-associated thrombosis compared with conventional anticoagulants.

TNF-α is able to induce apoptosis in tumor cells mainly by activating TNF receptor 1 (TNFR1)^45^. Upon activation, TNFR1 trigger apoptosis by recruiting and activating the apoptosis initiator caspase-8 through the adaptor Fas-associated death domain (FADD). Activated caspase-8 can either directly activate the executioner caspase-3 or indirectly activate caspase-9 and -3 via the mitochondrial apoptotic pathway. TNFR1 may also trigger an alternative form of programmed cell death, i.e., necroptosis, via a RIPK-dependent manner in the absence of caspase-8 activation. On the other hand, TNF-α/TNFR1 also induce survival signals by recruiting TNFR-associated factor 2 (TRAF-2), cIAP, and TAK1 that lead to activation of NF-κB, which acts as a negative regulator for the cytotoxic effect of TNF-α. In most types of cancer cells, the dominant outcome is cell survival, and thus resistant to TNF-α-induced apoptosis^46^. Consistent with previous studies, we observed no cytotoxic effect of TNF-α in NSCLC A549 and H1299 cells at the concentrations up to 100 ng/mL. On the other hand, 4HW at higher concentrations (above 1 μM) and longer exposure times (48 h) than that required to inhibit TNF-α-induced TF, caused synergistic cytotoxicity with TNF-α in NSCLC cells. The synergism of 4HW and TNF-α may have potential advantages in treating NSCLC in inflammatory microenvironments where TNF-α is highly expressed. Cell death caused by 4HW/TNF-α appears to be mediated by caspase-dependent apoptosis, as demonstrated by increased cleavage of caspases and PARP as well as decreased expression of anti-apoptotic proteins Bcl-xL, XIAP, and survivin. In contrast, necroptosis is unlikely to be involved in 4HW/TNF-α-induced cell death, because the latter was prevented by a pan-caspase inhibitor but not by necrostatin-1, which inhibits RIPK activity and thus necroptosis^47^.

NF-κB is the major transcription factor mediated TNF-α-induced inflammatory and survival signals^48^. TNF-α/TNFR1 stimulation initiates a signaling cascade leading to activation of IκB kinase (IKK), which in turn phosphorylates IκB and triggers its degradation via ubiquitin–proteasome system. IκB degradation liberates the NF-κB heterodimer p50/p65 to translocate to the nucleus where it turns on transcription of a number of genes involved in inflammation (such as IL-6, IL-8 and ICAM) and cell survival (such as anti-apoptotic IAP and Bcl-2 family proteins). In addition, TF is known also a target gene of NF-κB in response to TNF-α stimulation^49^. Hyperactivation of NF-κB has been observed in tumor tissues and positively correlated with poor prognosis of lung cancer patients^50^, as well as the higher risk of venous thromboembolism^51^. Our present results also support that NF-κB is a master switch for both inflammation-induced coagulation and cell survival in NSCLC. In this study, 4HW exhibited inhibitory effect on TNF-α–induced NF-κB signaling, as evident by reduced p65 nuclear accumulation, transcriptional activity, and expression of NF-κB-responsive genes, such as anti-apoptotic proteins and TF. By inhibiting NF-κB, 4HW can reverse the pro-coagulant effect of TNF-α but enhance its cytotoxic effect. It is worth noticing that the mechanism by which 4HW inhibits TNF-α-induced NF-κB signaling appears to be different from other NF-κB inhibitors, since it does not prevent the early events such as IκB degradation and p65 translocation into the nucleus; instead, 4HW reduces p65 nuclear accumulation by accelerating p65 export from the nucleus. Many factors such as histone deacetylase-3, annexin A6, and high mobility group protein N2 might regulate p65 nuclear retention time^52–54^, so further studies are required to investigate how 4HW affects this process.

In conclusion, we have demonstrated that the inflammatory cytokine TNF-α induces TF expression as well as procoagulant activity in NSCLC cells, and this effect can be potently inhibited by a natural compound, 4HW. Moreover, combination of 4HW and TNF-α leads to synergistic cytotoxicity against NSCLC cells. These results suggest that 4HW may have potential application for treating inflammation-derived cancer progression and cancer-associated hypercoagulable states.

## Methods

### Reagents

4HW was isolated from *P. peruviana* with a purity of 98% according to the method described previously^28^. Human recombinant TNF-α was purchased form R&D Systems, Inc. (Minneapolis, MN, USA). Ro 106-9920 was purchased from Santa Cruz Biotechnology (Santa Cruz, CA, USA). Leptomycin B, Z-VAD(OMe)-FMK, necrostatin-1, and wortmannin were purchased from Cayman Chemical (Ann Arbor, MI, USA). 3-(4,5-dimethylthiazol-2-yl)-2,5-diphenyltetrazolium bromide (MTT) was purchased from Sigma-Aldrich (St. Louis, MO, USA). Fetal bovine serum (FBS) was purchased from Thermo Fisher Scientific (Waltham, MA, USA).

### Cell culture

Human lung adenocarcinoma epithelial cell lines H1299 and A549 cells were obtained from American Type Culture Collection (ATCC, USA). Human embryonic kidney cells (HEK) 293 T was purchased from Bioresource Collection and Research Center (BCRC, Hsinchu, Taiwan). All of the three cell lines were maintained in Dulbecco’s Modified Eagle’s Medium (DMEM)/F12 (Thermo Fisher Scientific) supplemented with 10% FBS and 1% penicillin/streptomycin, and rested in a humidified incubator at 37 °C with 5% CO_2_.

### Cell viability assay

Cell viability was assessed by MTT assay as previously described^55^. Cancer cells were seeded in a 96-well plate (1 × 10^4^ cells/well) in triplicates and treated with drugs in 5% CO_2_ at 37 °C for 24 h. At the end, culture medium was removed and MTT (0.5 mg/mL) was added to each well. Cells were further incubated at 37 °C for 2 h. The formazan crystals were solubilized with DMSO and the absorbance was read at 550 nm.

### Real-time reverse transcription polymerase chain reaction (RT-PCR)

Total RNA was extracted from cancer cells by GeneMark Total RNA Miniprep Purification Kit (GMbiolab Co, Ltd., Taiwan). The mRNA templates were converted into a complementary DNA (cDNA) by using a High-Capacity cDNA Reverse Transcription Kit (Applied Biosystems, Foster City, CA, USA). The cDNA samples were mixed with KAPA SYBR FAST qPCR Master and primers. The polymerase chain reaction was carried out and analyzed by using Applied Biosystems StepOne Real-Time PCR System. Primers used in this study are listed below: TF forward primer 5′-GCCAGGAGAAAGGGGAAT-3′ and reverse primer 5′-CAGTGCAATATAGCATTTGCAGTAGC-3′; β-actin forward primer 5′-TCACCCACACT GTGCCCATCTACGA-3′ and reverse primer 5′-CAGCGGAACCGCTCATTGCCAATGG-3′. Every single experiment was performed in triplicates. The fold changes in gene expression levels were calculated with normalization to β-actin values by using 2^−ΔΔCt^ comparative cycle threshold method.

### Western blot analysis

The protein expression was determined by Western blot analysis as previously described^55^. Cells were harvested and lysed with lysis buffer (50 mM Tris–HCl pH = 7.4, 150 mM NaCl, 1 mM EDTA, 1 mM EGTA, 1% Triton X-100) containing protease inhibitor and phosphatase inhibitor (Roche Applied Science, Penzberg, Germany) for 15 min on ice. Protein concentrations were determined by Bradford protein assay (Bio-Rad, Richmond, CA, USA). Equal amounts of protein (10–20 μg) from cell lysates were separated by SDS-PAGE and transferred to nitrocellulose membranes. The membrane was blocked with 5% non-fat milk and incubated with specific primary antibody overnight. Then, the membrane was incubated with horseradish peroxidase (HRP)-conjugated secondary antibody for 1 h. Finally, the membrane was probed by the chemiluminescence detection kit (Millipore, Bedford, MA, USA) and visualized with a LAS-4000mini imaging system (Fujifilm, Tokyo, Japan). Antibodies against various proteins were listed as follows: TF antibodies (#4509 and 4503) were purchased from BioMedica Diagnostics Inc. (Windsor, Canada). PARP (sc-8007) and β-Actin (sc-8432) antibodies were purchased from Santa Cruz Biotechnology (Santa Cruz, CA, USA). NF-кB p65 (#4764), IкBα (#4814), GAPDH (#2118), caspase 3 (#9662), caspase 8 (#9746), caspase 9 (#9502), XIAP (#2045), and Bcl-xL (#2764) antibodies were purchased from Cell Signaling Technology Inc. (Danvers, MA, USA).

### Tilt tube plasma clotting assay

Human blood was isolated from healthy human volunteers and anticoagulated with sodium citrate. This study was approved by the Institutional Review Board of Kaohsiung Medical University; all experiments were performed in accordance with relevant guidelines and regulations, and informed consent was acquired from all volunteers. Plasma clot induced by cancer cells was measured as previously described^55^. In brief, 200 μL of cancer cell suspension (2.5 × 10^5^ cells/mL in phosphate-buffered saline, PBS) was mixed with an equal volume of human plasma in a test tube, then 200 μL of CaCl_2_ solution (25 mM in normal saline) was added to trigger plasma clot at 37 °C. Plasma clotting was observed visually and checked every 5 s. The clotting time was recorded when plasma formed a semisolid gel that did not flow during tube tilting.

### TF/factor VII/Xa activity assay

TF-dependent procoagulant activity was measured by amidolytic assay as previously described^55^. Cancer cells were seeded in a 96-well plate (5 × 10^3^ cells/well) and incubated overnight in 5% CO_2_ at 37 °C. Cells were pretreated with drugs for 1 h, followed by treating with TNF-α for 24 h. After removal of culture medium, cells were washed twice with PBS, and incubated with 10 nM human factor VIIa and 175 nM factor X (Haematologic Technologies, Inc., Essex Junction, VT, USA) in Tris-buffered saline (TBS, containing 5% BSA and 25 mM CaCl_2_) at 37 °C for 10 min. The reaction was terminated by adding the stop solution (TBS with EDTA 25 mM). After then, Spectrozyme factor Xa substrate (1 mM, Sekisui Diagnostics, Lexington, MA, USA) was added, and the absorbance was read at 405 nm in the kinetic mode. The end point of the linear portion of the absorbance curve was recorded. The results of TF activity were normalized to cell viability and relative to untreated controls.

### Flow cytometry and annexin V/propidium iodide (PI) staining

Apoptotic and necrotic cells were detected by Dead Cell Apoptosis kit (Invitrogen, Carlsbad, CA, USA) USA) according to manufacturer’s protocol. Cancer cells treated with drugs were harvested and resuspended in annexin V binding buffer, and incubated with Alexa Fluor 488-Annexin V and PI for 15 min. Cell samples were then analyzed by BD LSR II flow cytometer (BD Biosciences-US, San Jose, CA, USA).

### Immunofluorescence microscopy

Cancer cells (1 × 10^4^ cells) were seeded in the glass coverslips and incubated in 5% CO_2_ at 37 °C overnight. After treatment with drugs, cells were washed with PBS and fixed by 4% paraformaldehyde for 20 min at room temperature. The fixed cells were permeabilized with 0.05% Triton-X 100 in PBS for 20 min, and then blocked with SuperBlock (Thermo Fisher Scientific) in 4 °C overnight. After blocking, cells were incubated with NF-кB primary antibody for overnight and then with Alexa Fluor 594-Goat anti-mouse secondary antibody (Jackson ImmunoResearch Inc., West Grove, PA, USA) and 4′,6-diamidino-2-phenylindole (DAPI, 1 mg/mL) for 1 h. The stained cells were washed and sealed with coverslips and the immunofluorescence images were acquired by Cell^R Xcellence fluorescence microscope system (Olympus, Japan).

### Knockdown of XPO1 with shRNA

The XPO1 shRNA plasmid purchased from the National RNAi Core Facility (Academia Sinica, Taipei, Taiwan) was used to transfect NSCLC cells according to the method described previously^56^. Cancer cells were seeded in a 6-well plate (2.5 × 10^5^ cells/well) and incubated in 5% CO_2_ at 37  °C until they reached 60—80% confluence. Plasmid DNA (1 μg) and Lipofectamine 2000 (3 μL; Invitrogen, Carlsbad, CA, USA) were mixed in Opti-MEM I Medium (200 μL; Thermo Scientific, Waltham, MA, USA), and incubated at room temperature for 20 min. Cells were then incubated with this mixture at 37 °C and 5% CO_2_ for 24 h. After transfection, the medium was replaced with fresh medium supplemented with 10% fetal bovine serum.

### NF-κB activation assay

The activation of NF-κB was measured by using NF-κB-luciferase-based promoter-reporter assay. Human embryonic kidney (HEK) 293 T cells or NSCLC H1299 cells transfected with pGL4.32[luc2P/NF-κB-RE/Hygro] vector (Promega Corporation, USA) were seeded in 96-well microplates and treated with drugs in triplicate, and then incubated in 5% CO_2_ at 37 °C for 6 h. After then, the culture medium was removed and the luciferin reagent (Bright-Glo Luciferase Assay System, Promega, USA) was added into each well and incubated at room temperature for 10 min, the luminescence was measured in a luminometer.

### Statistical analysis

Data were analyzed using Prism 5 (Graphpad Software, Inc., San Diego, CA, USA) and presented as mean ± SEM. Significance was estimated by using one- or two-way analysis of variance (ANOVA) tests.

## Supplementary Information

Supplementary Information
