# Supplementary material for: Golden berry 4β-hydroxywithanolide E prevents tumor necrosis factor α-induced procoagulant activity with enhanced cytotoxicity against human lung cancer cells
Source: Sci Rep. 2021 Feb 25;11:4610. doi: 10.1038/s41598-021-84207-8 (PMC7907079; doi:10.1038/s41598-021-84207-8)
Supplement: Supplementary file 1 — Supplementary Information [file 41598_2021_84207_MOESM1_ESM.docx]

**Supplementary data**

**Golden berry 4β-hydroxywithanolide E prevents tumor necrosis factor α-induced procoagulant activity with enhanced cytotoxicity against human lung cancer cells**

Kan-Yen Hsieh^1^, Ju-Ying Tsai^1^, Ya-Han Lin^1^, Fang-Rong Chang^1,2^, Hui-Chun Wang^1,2^, Chin-Chung Wu^1,2*^

^1^ Graduate Institute of Natural Products, Drug Development and Value Creation Research Center, Kaohsiung Medical University, Kaohsiung, Taiwan

^2^ Department of Medical Research, Kaohsiung Medical University Hospital, Kaohsiung, Taiwan

^*^Corresponding author. E-mail address: [ccwu@kmu.edu.tw](mailto:ccwu@kmu.edu.tw) ; TEL: 886-7-3121101 ext. 2162


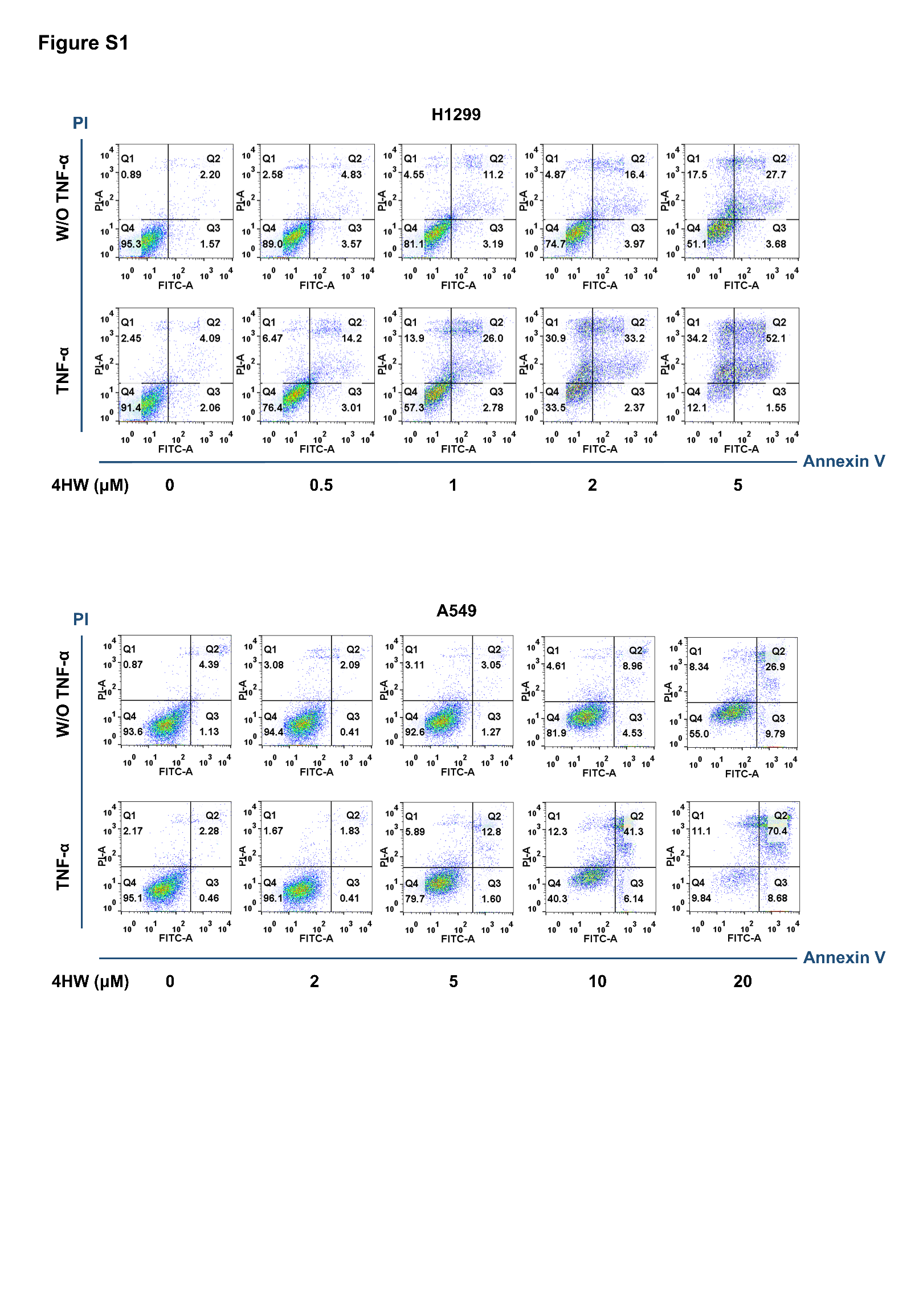


**Figure S1.** Representative flow cytometry data for Figure 3C.
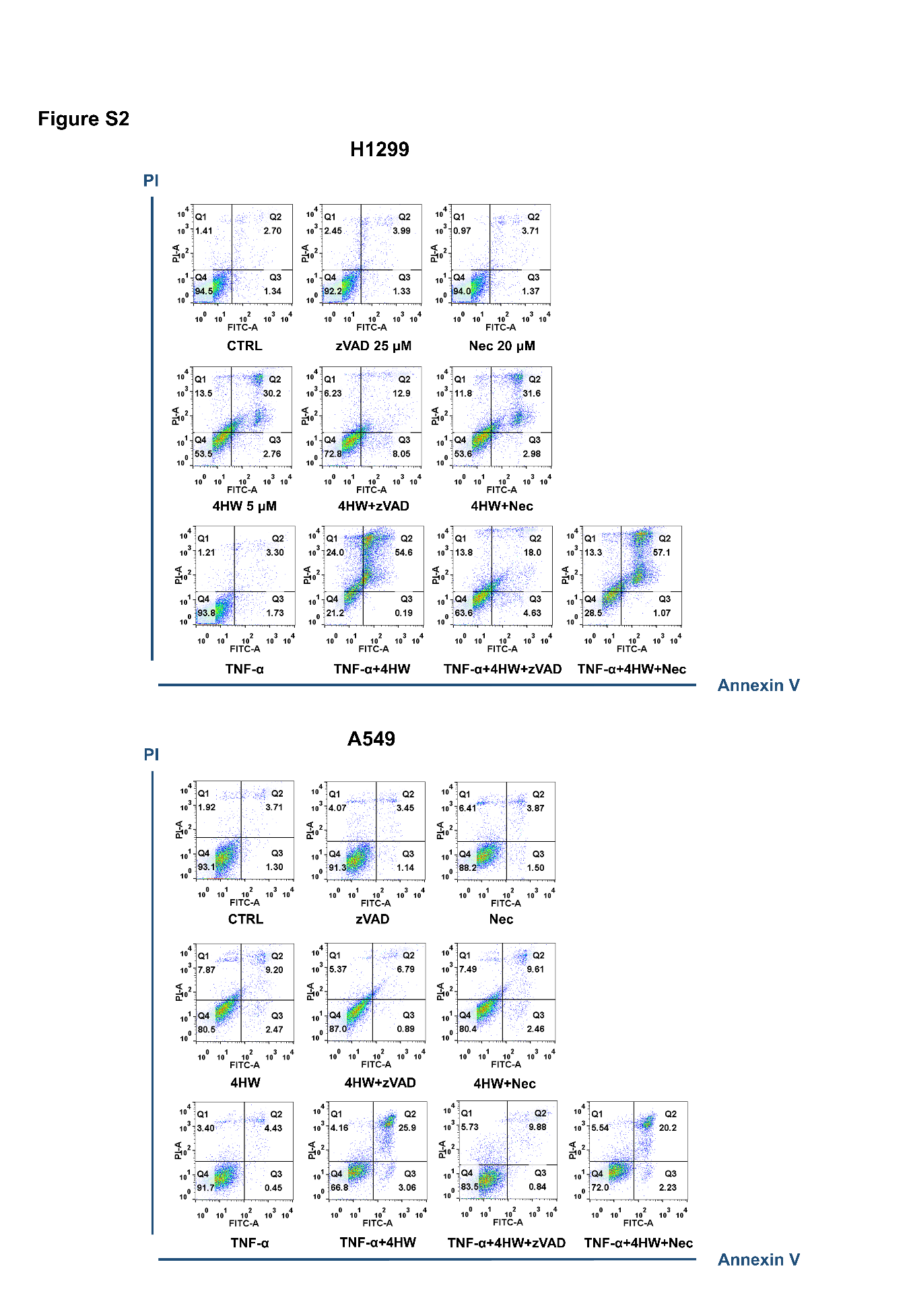


**Figure S2.** Representative flow cytometry data for Figure 4B.

**
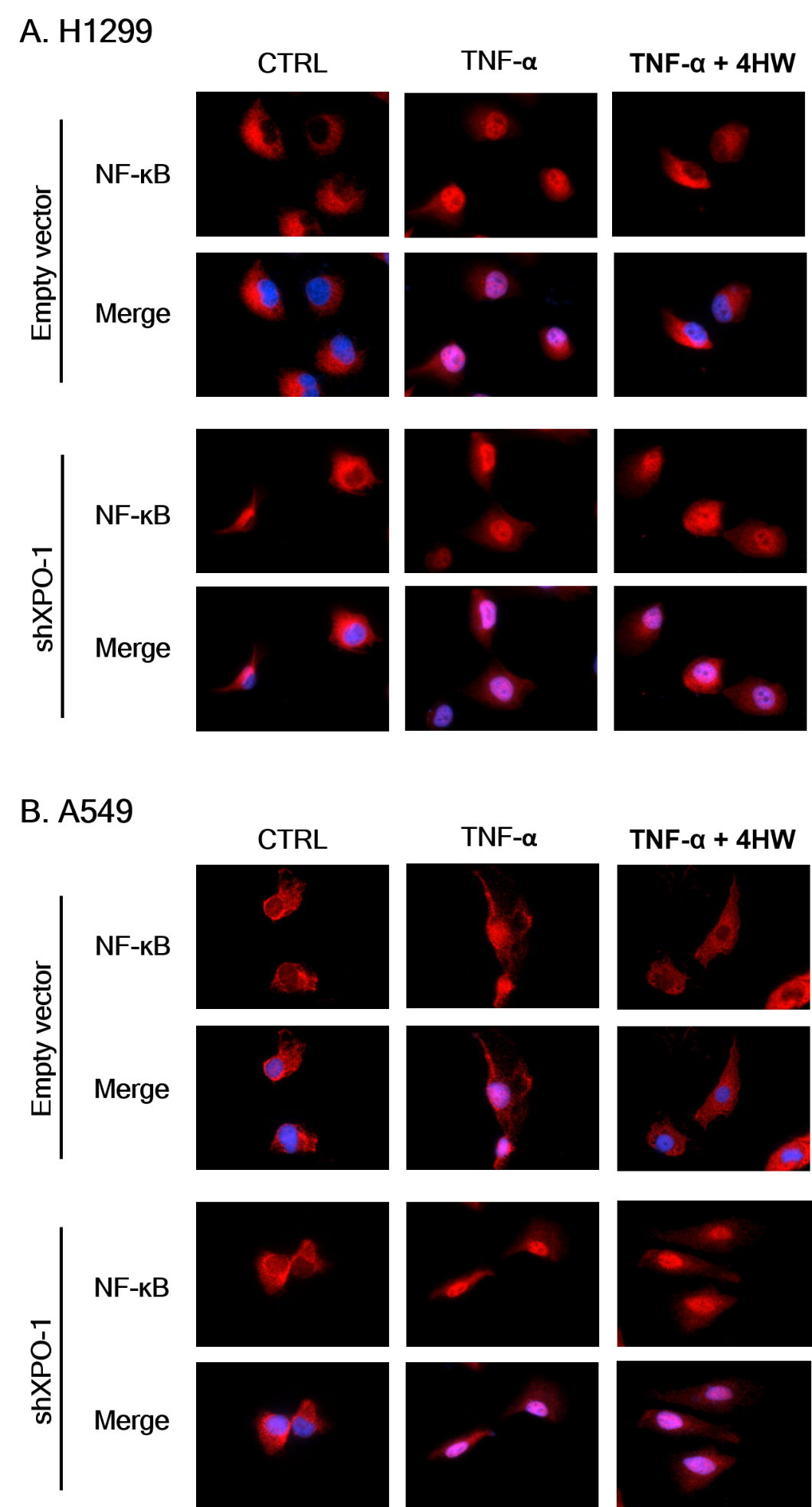
**

**Figure S3. Knockdown of XPO1 blunts the effect of 4HW on TNF-α-induced NF-κB nuclear accumulation.** In H1299 and A549 cells, XPO1 was silenced using shRNA as described in the Method section. These cancer cells were treated with 4HW (1 μM for H1299, 2 μM for A549) or DMSO (vehicle control), and then stimulated with TNF-α (20 ng/mL) for 4 hours. Nuclear translocation of NF-κB p65 (red) was determined by immunofluorescence microscopy. Nuclei were counterstained with DAPI (blue). The images were overlapped (Merge) to determine translocation.**Unedited and replicate images of western blots**


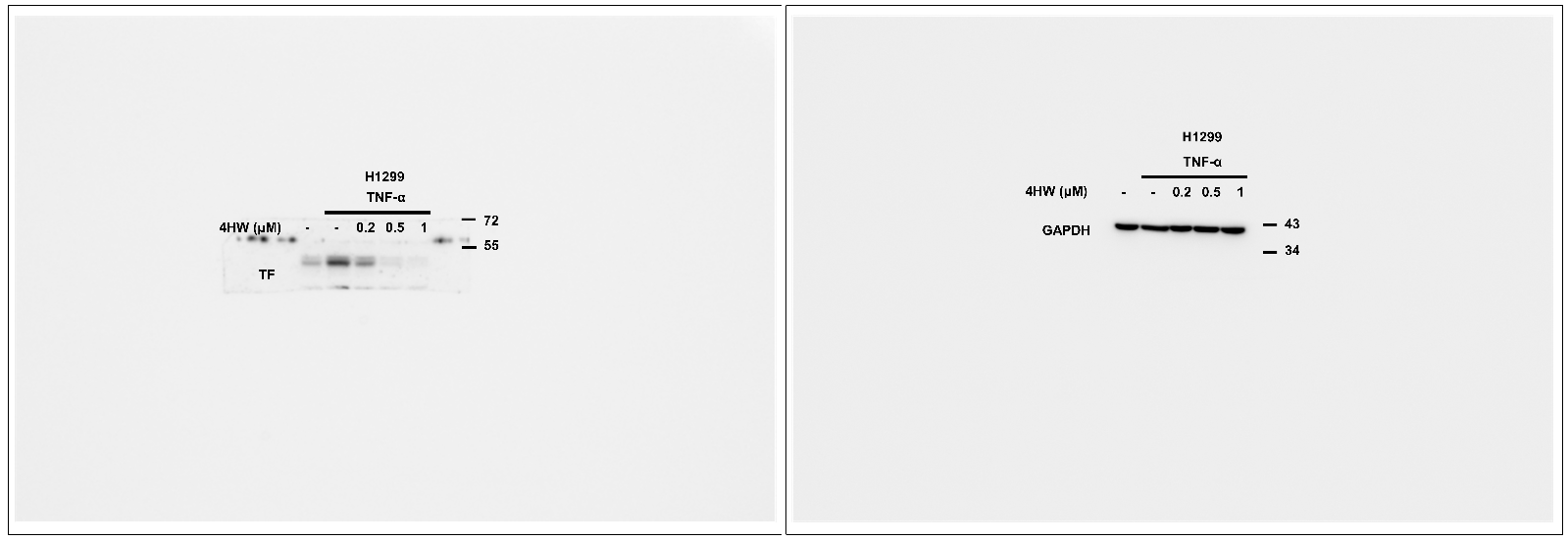

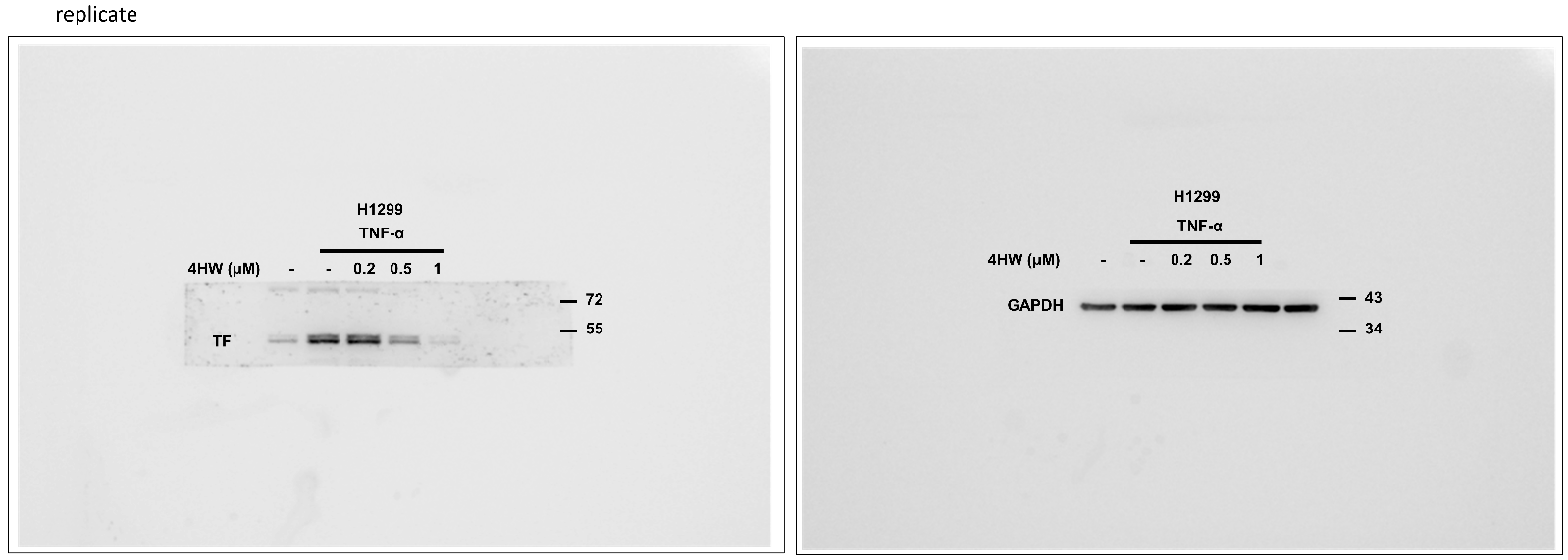

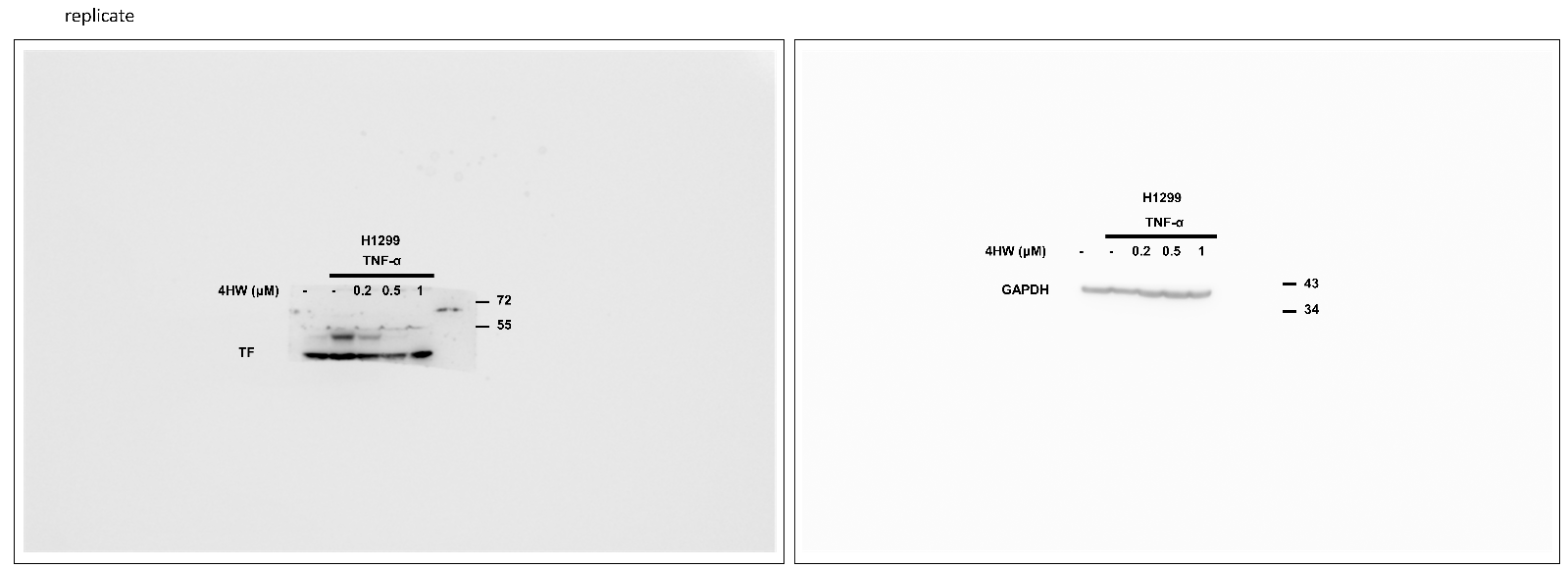


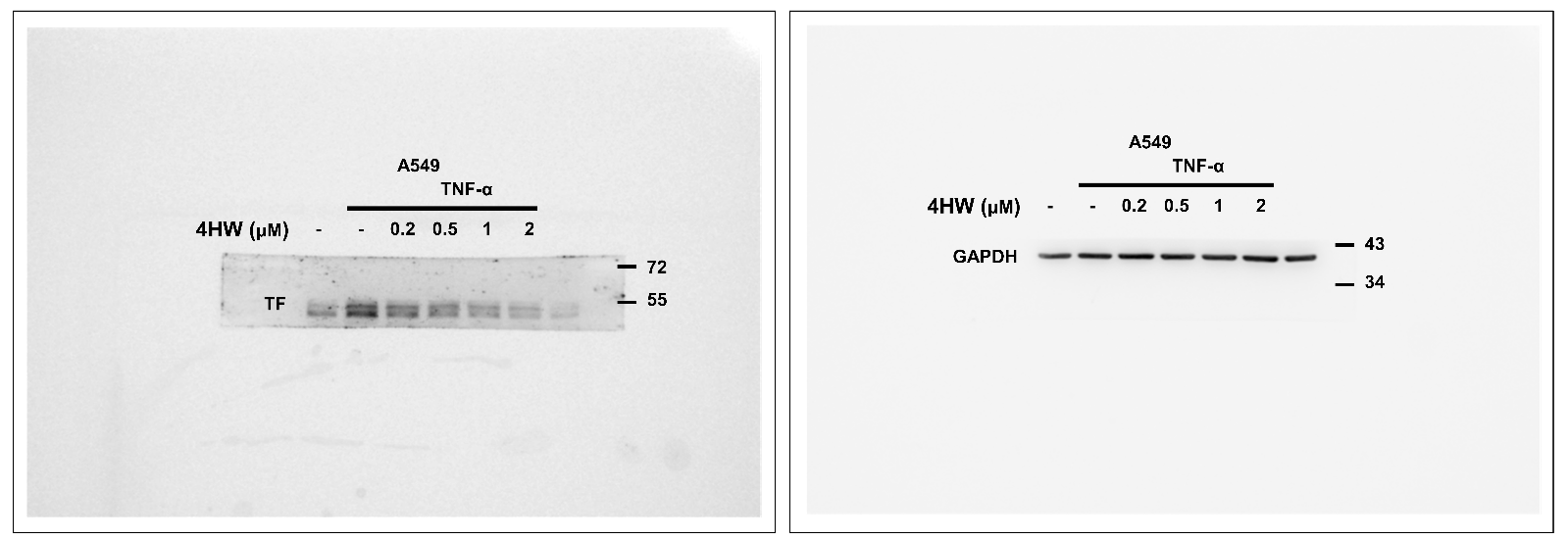

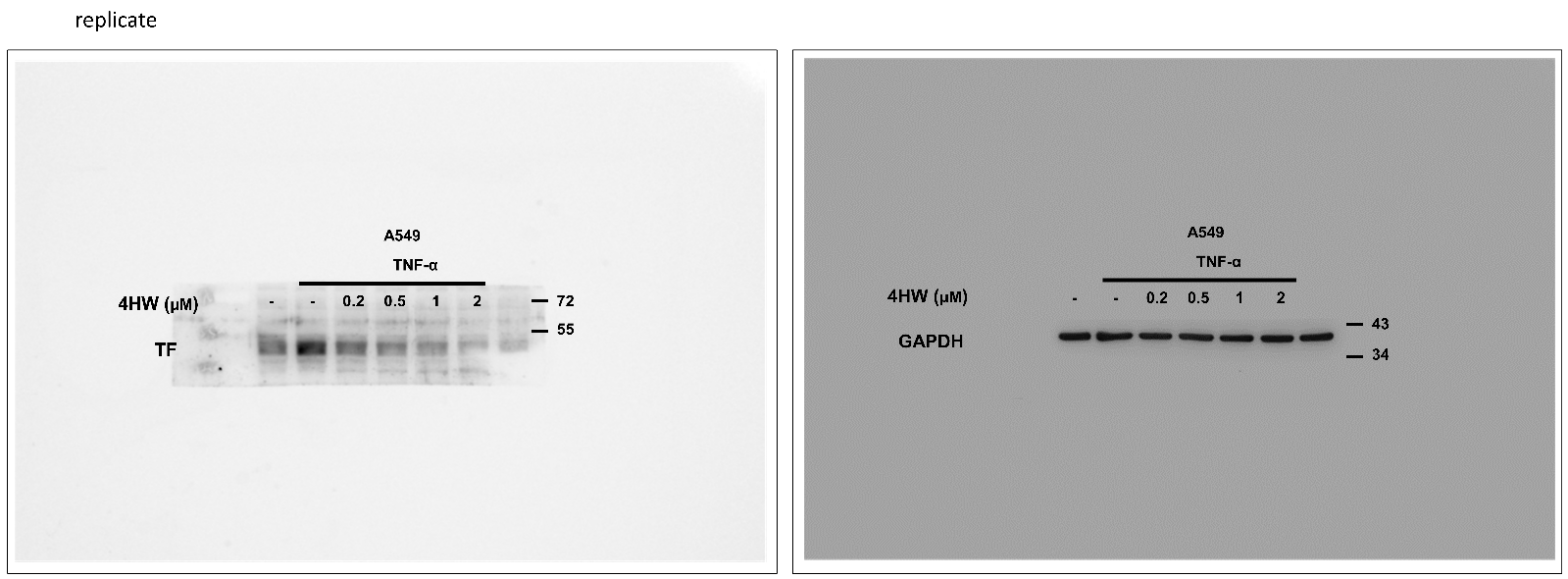

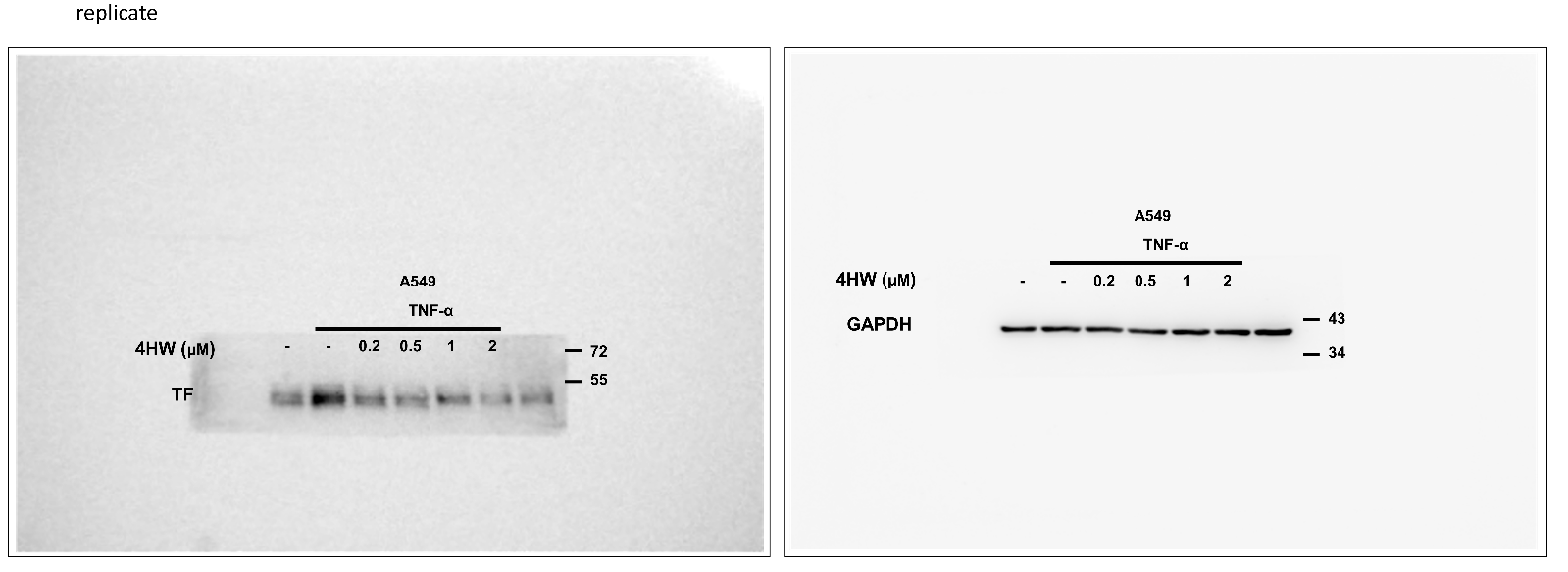


**Unedited blots and replicates of TF and GAPDH in the figure 1.** Blots were prepared from the same nitrocellulose membrane and cut prior to hybridization with antibodies. Some lanes irrelevant to this study in the original blot were removed.


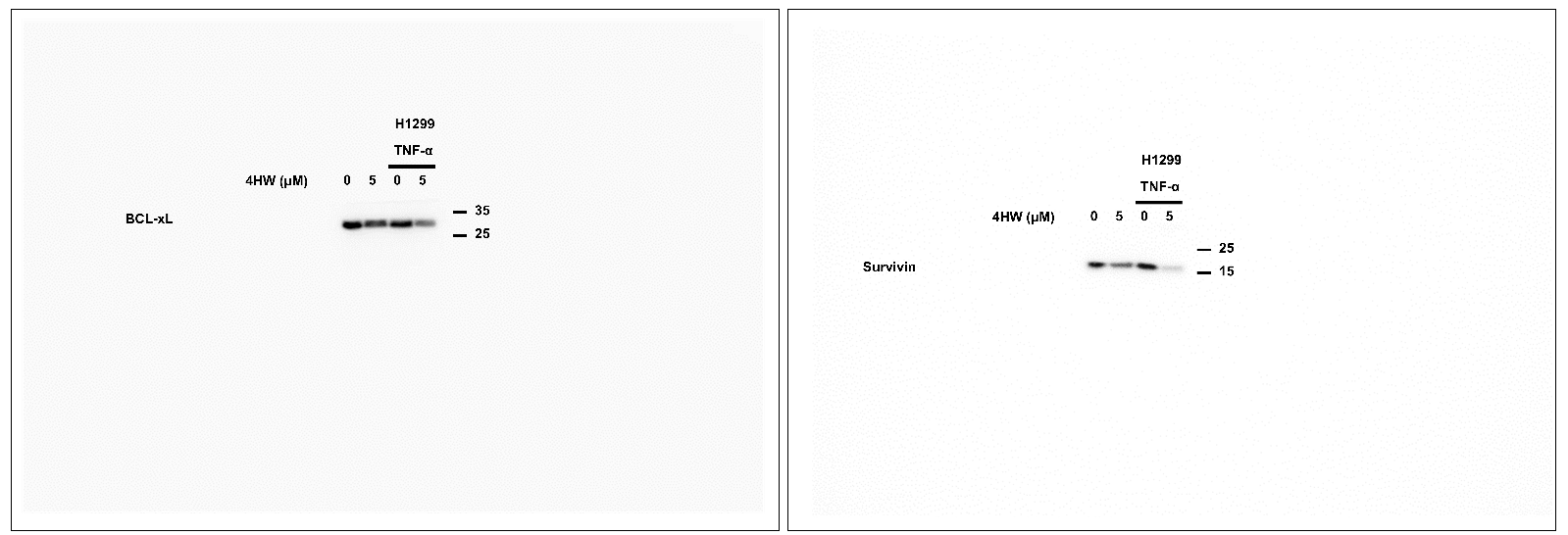

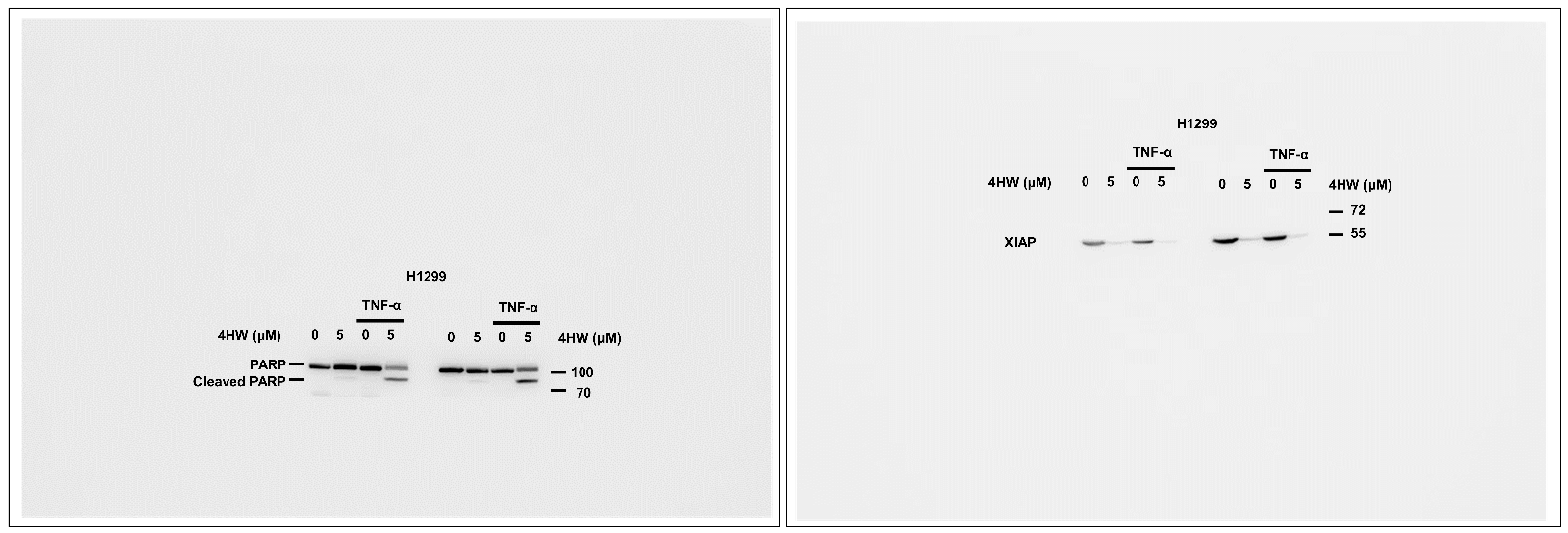

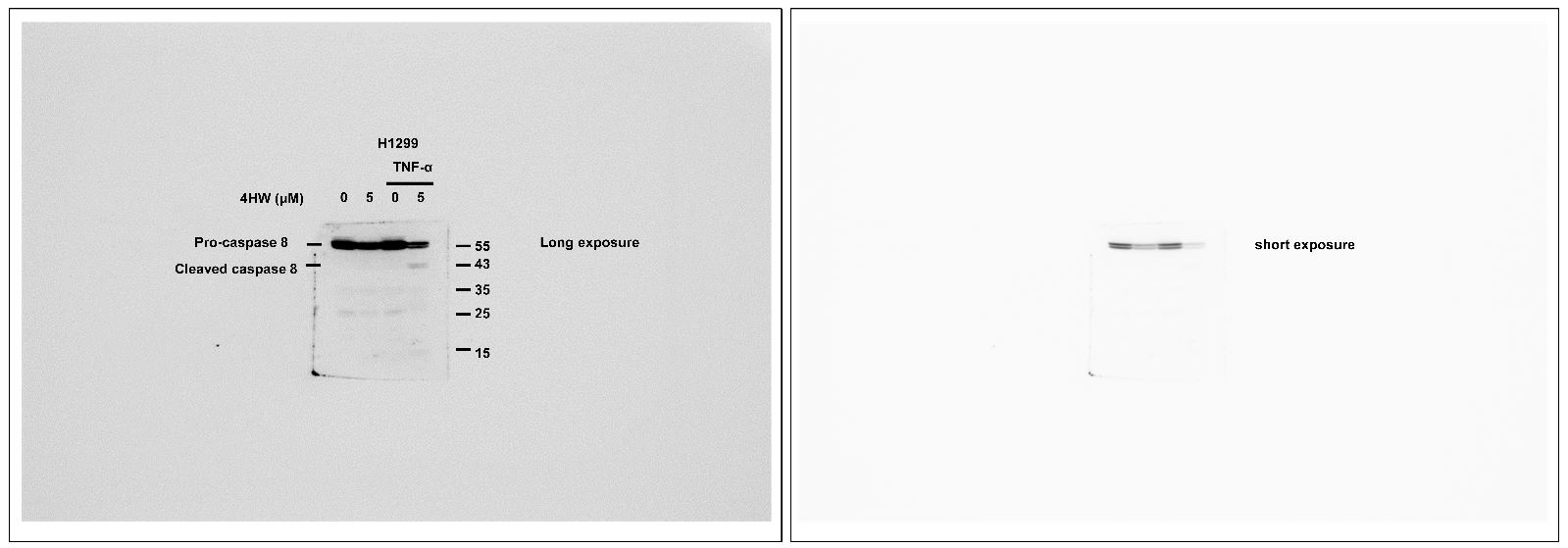

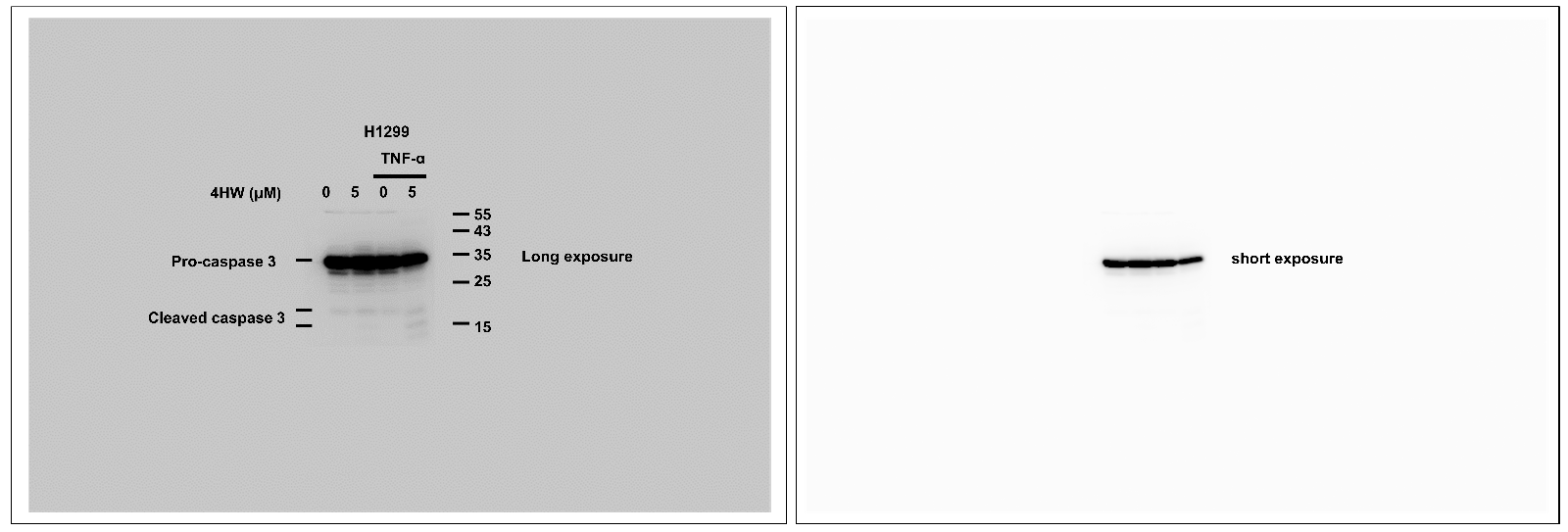

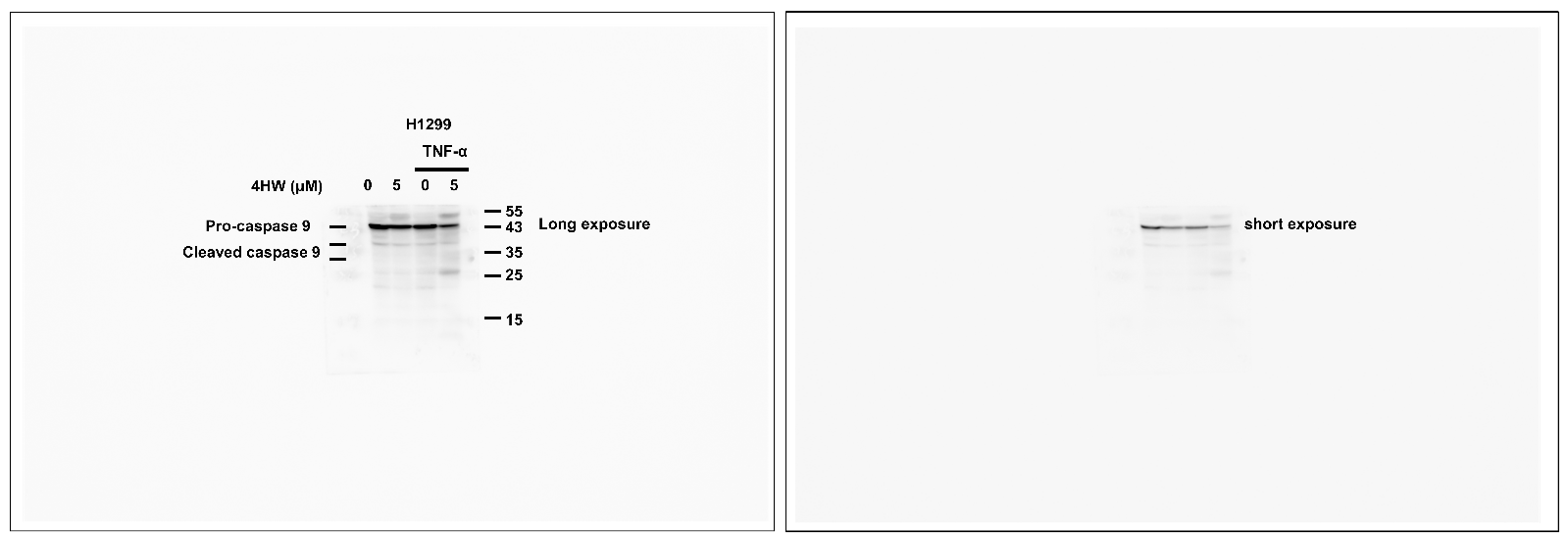

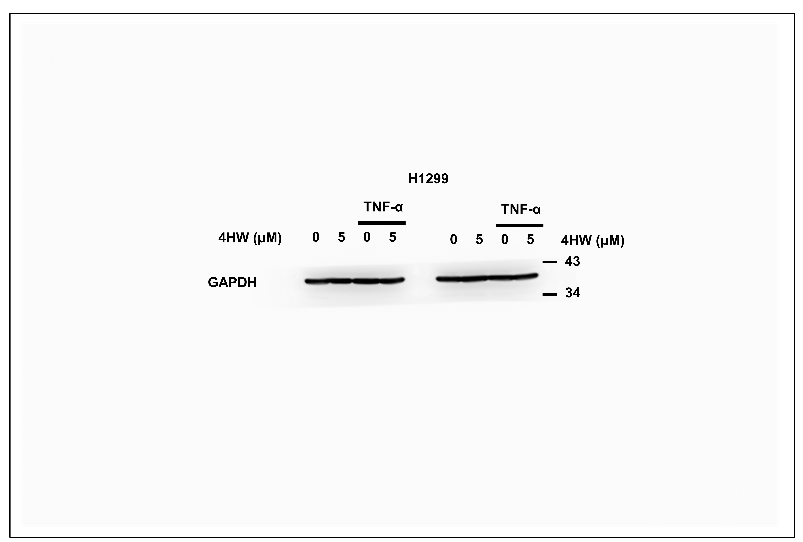


**Unedited blots of apoptosis-related biomarkers in the figure 4 (H1299).** Blots of BCL-xL, survivin, XIAP, PARP, and GAPDH were cut prior to hybridization with antibodies. Low-exposure image of caspase 3, 8, and 9 was shown next to the high-contrast blots.


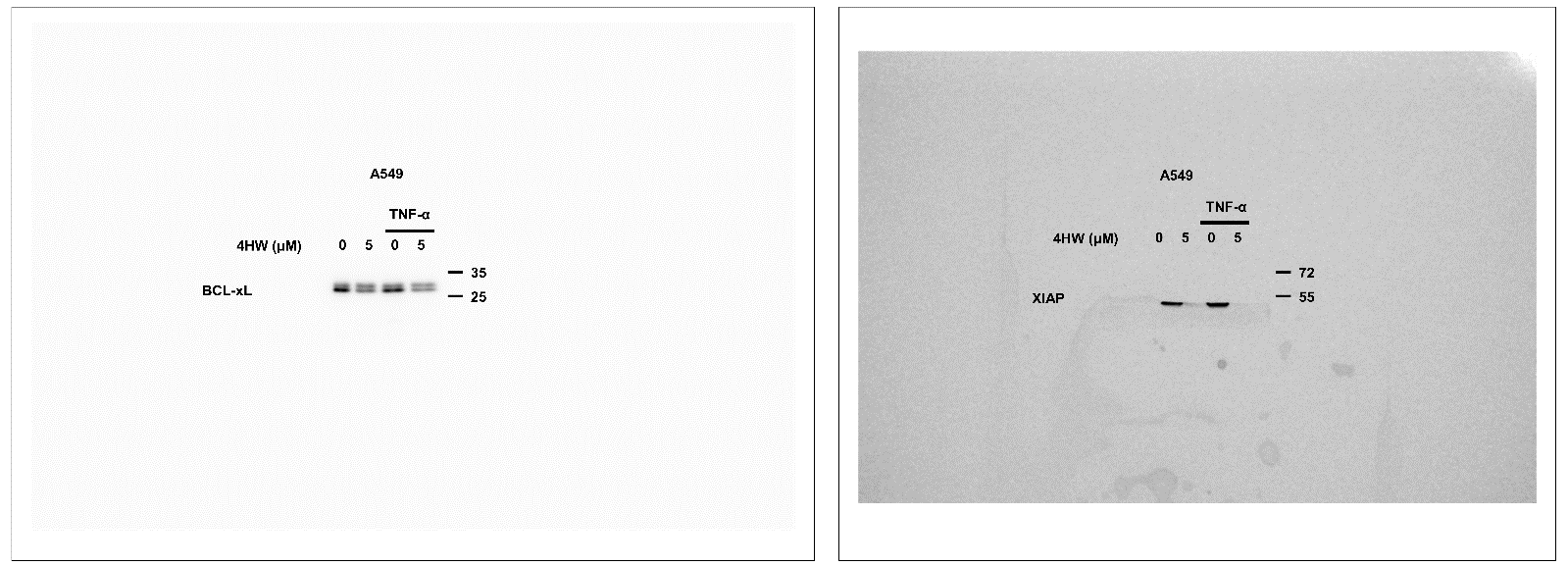

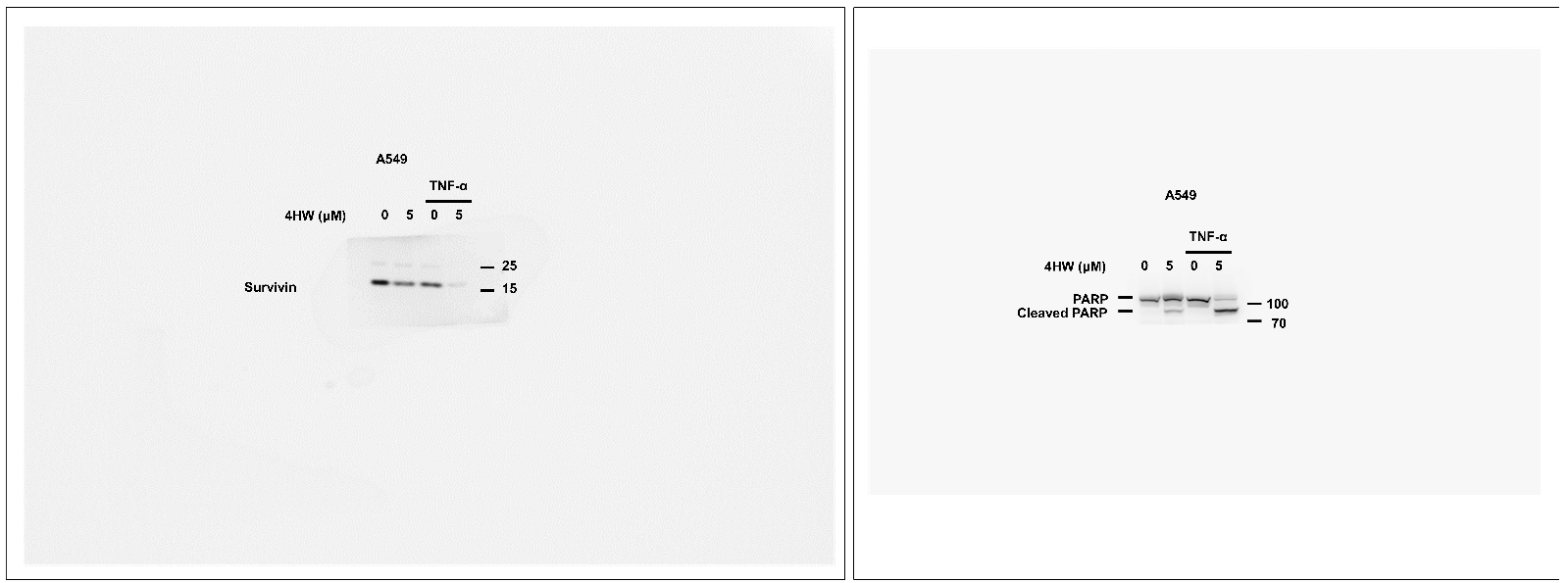

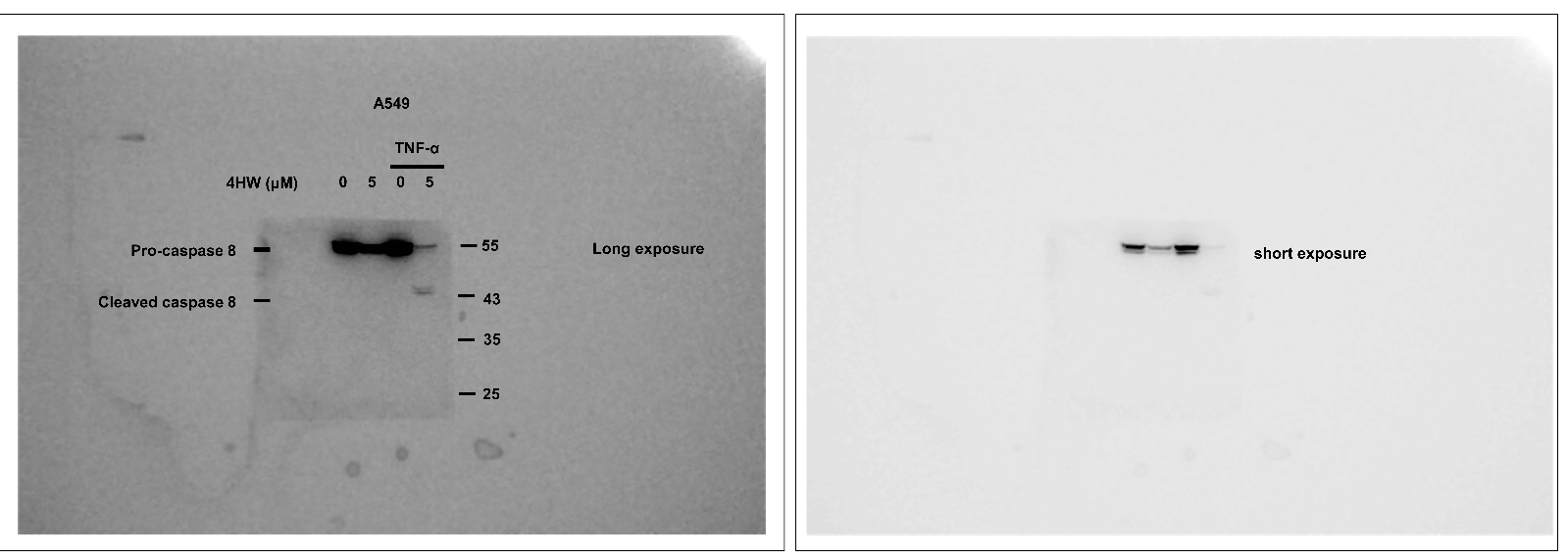

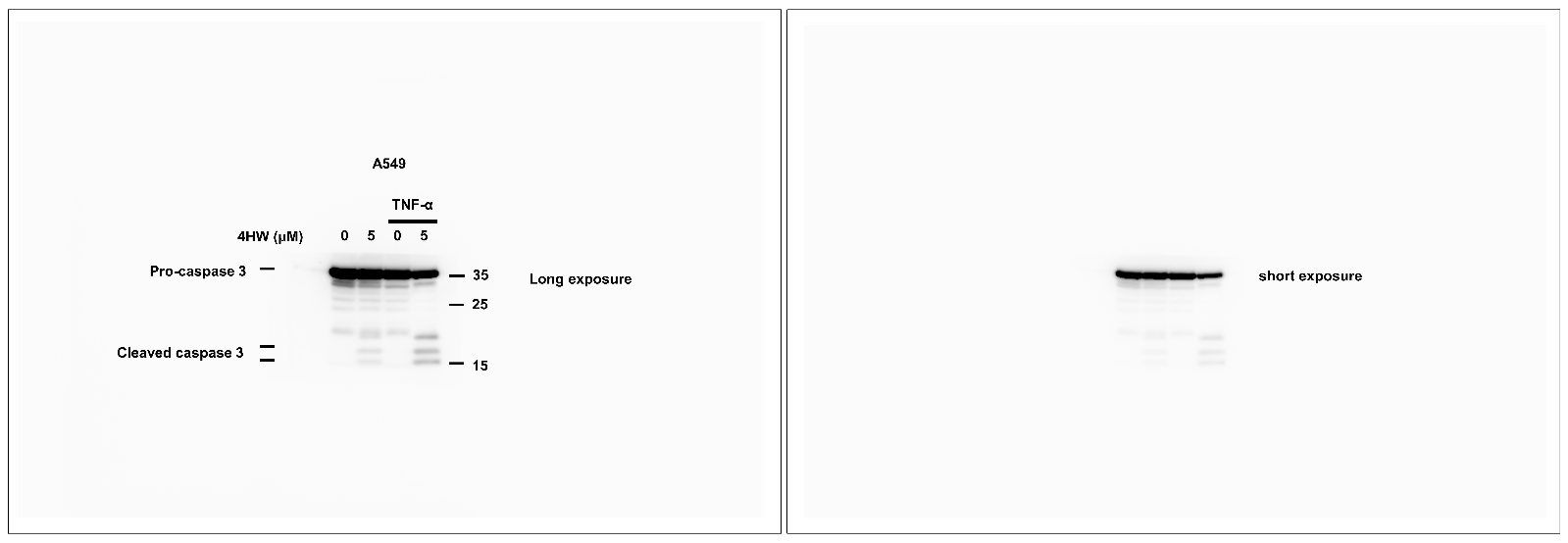

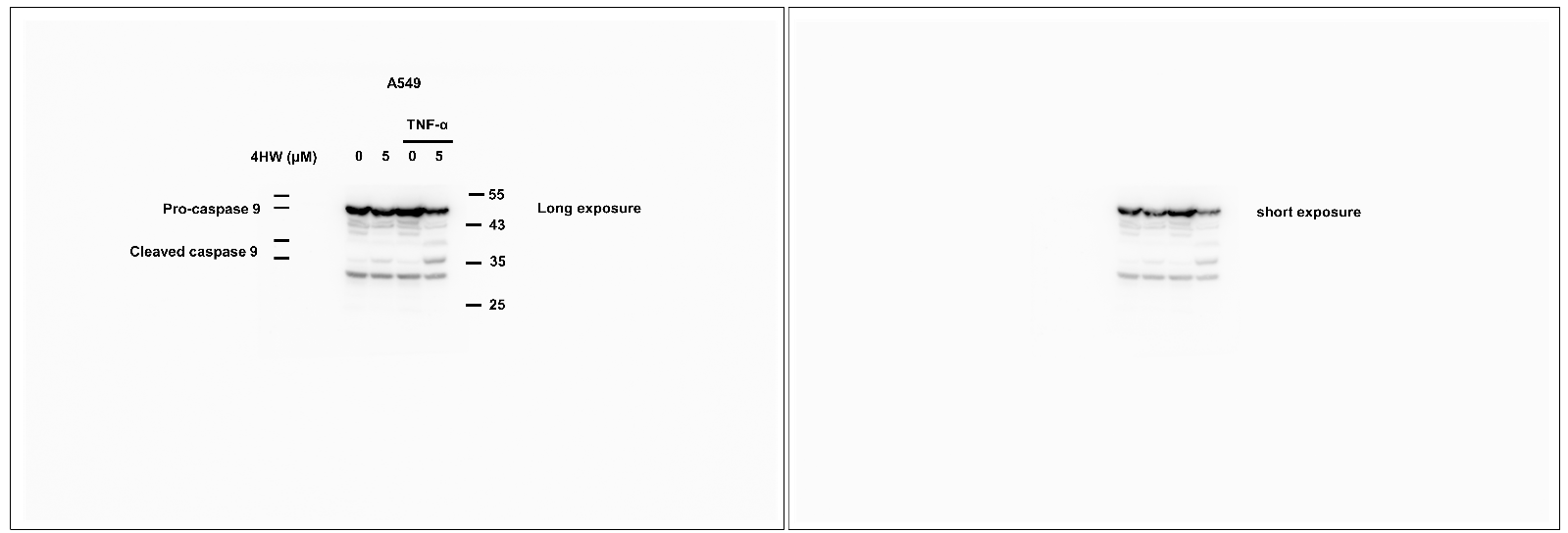

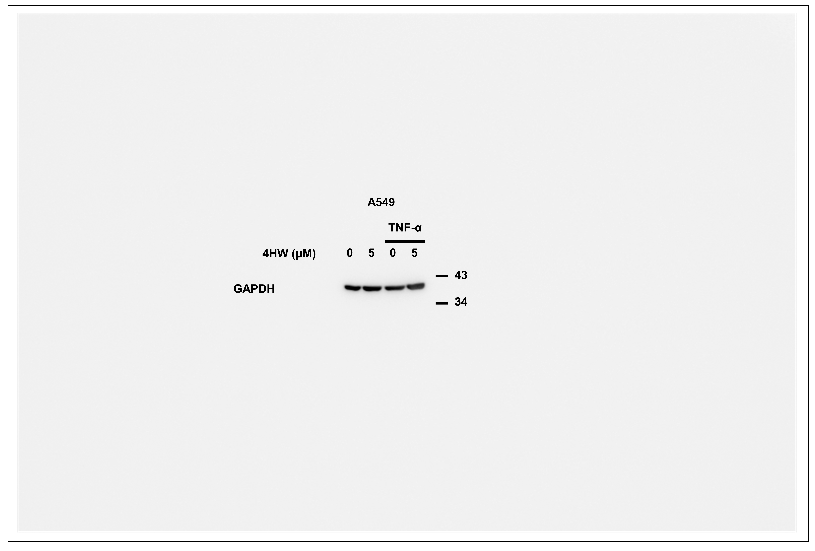


**Unedited blots of apoptosis-related biomarkers in the figure 4 (A549).** Blots of BCL-xL, survivin, XIAP, PARP, and GAPDH were cut prior to hybridization with antibodies. Low-exposure image of caspase 3, 8, and 9 was shown next to the high-contrast blots.


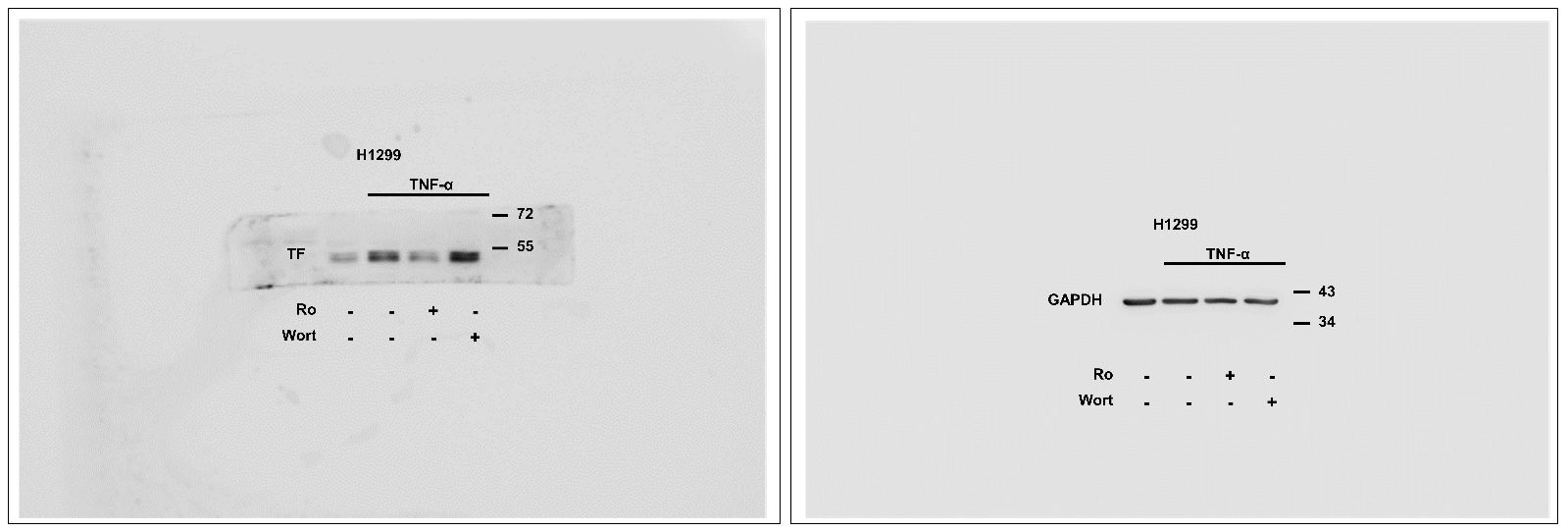

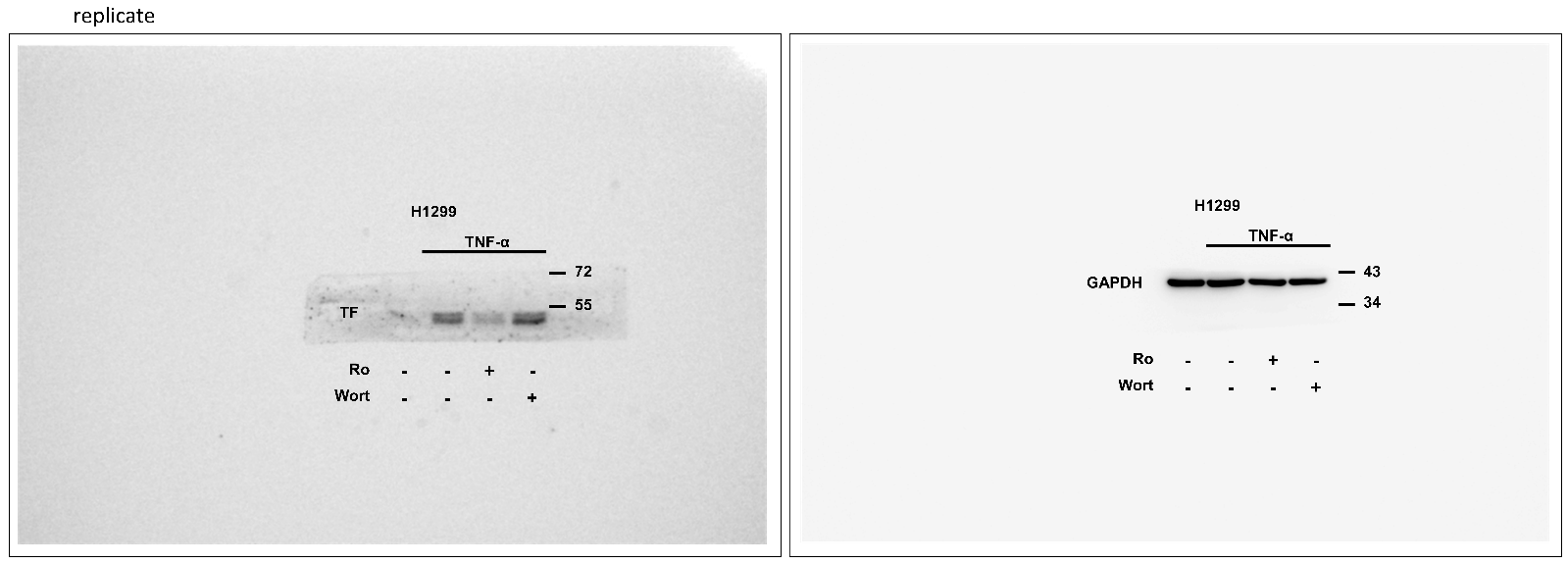

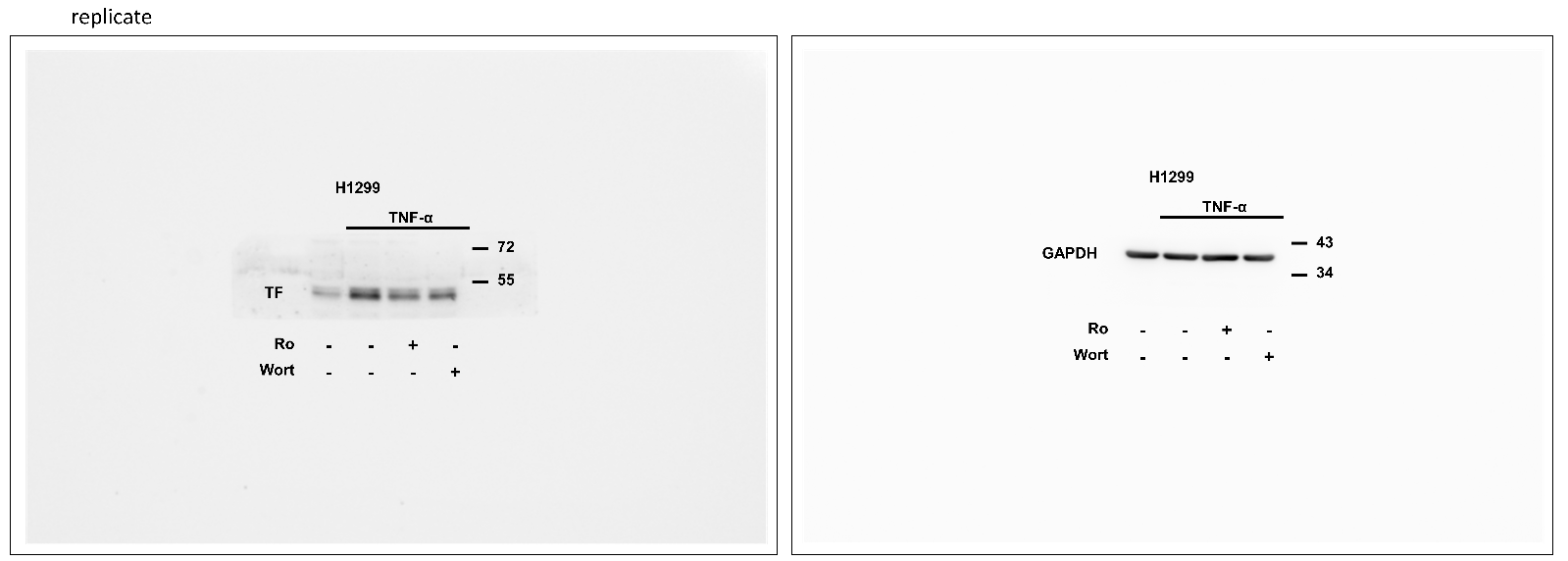

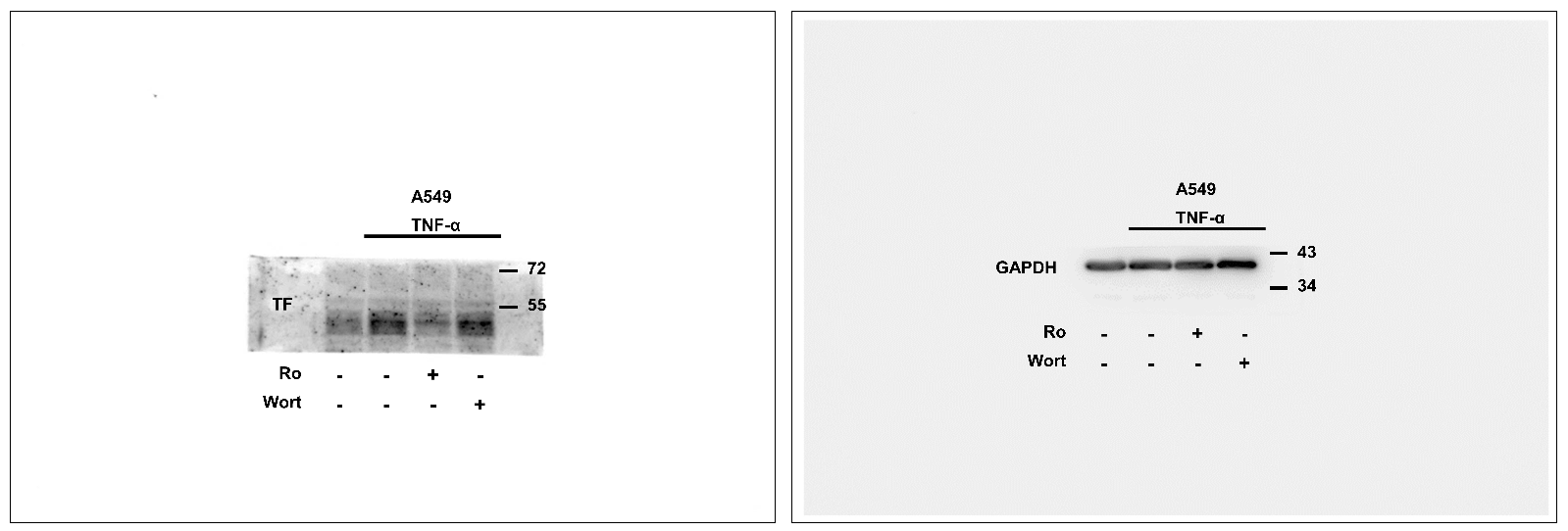

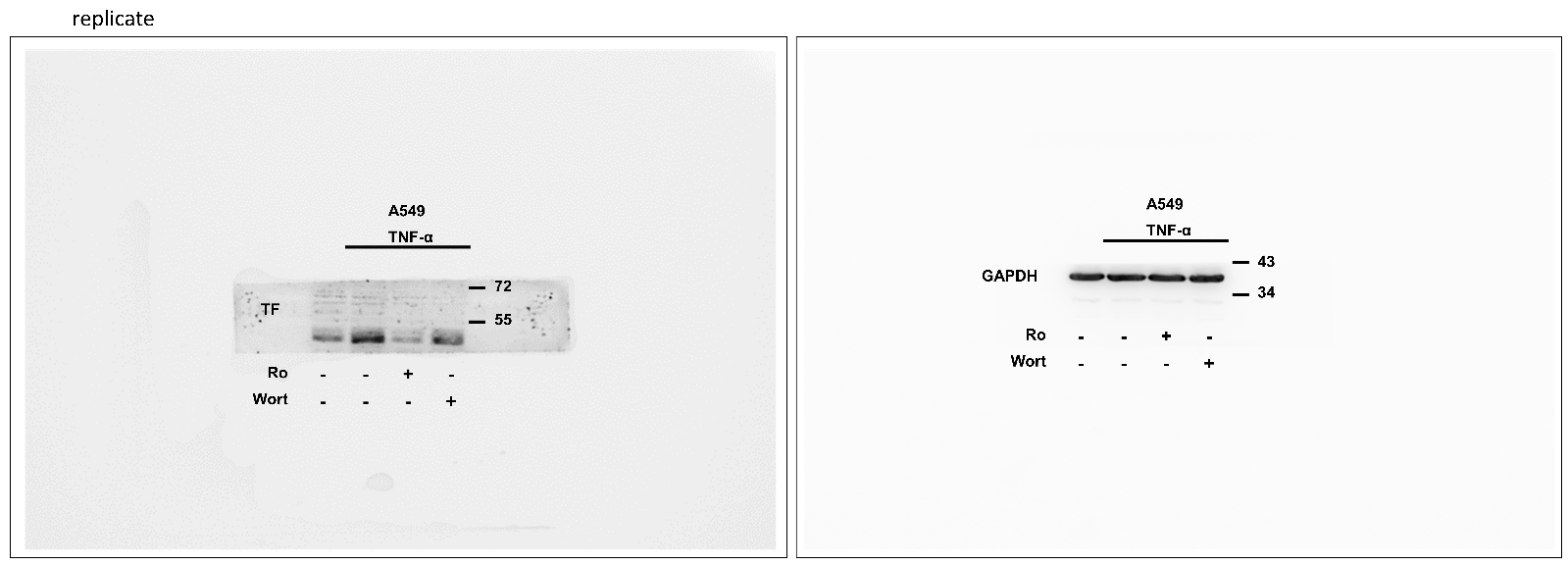

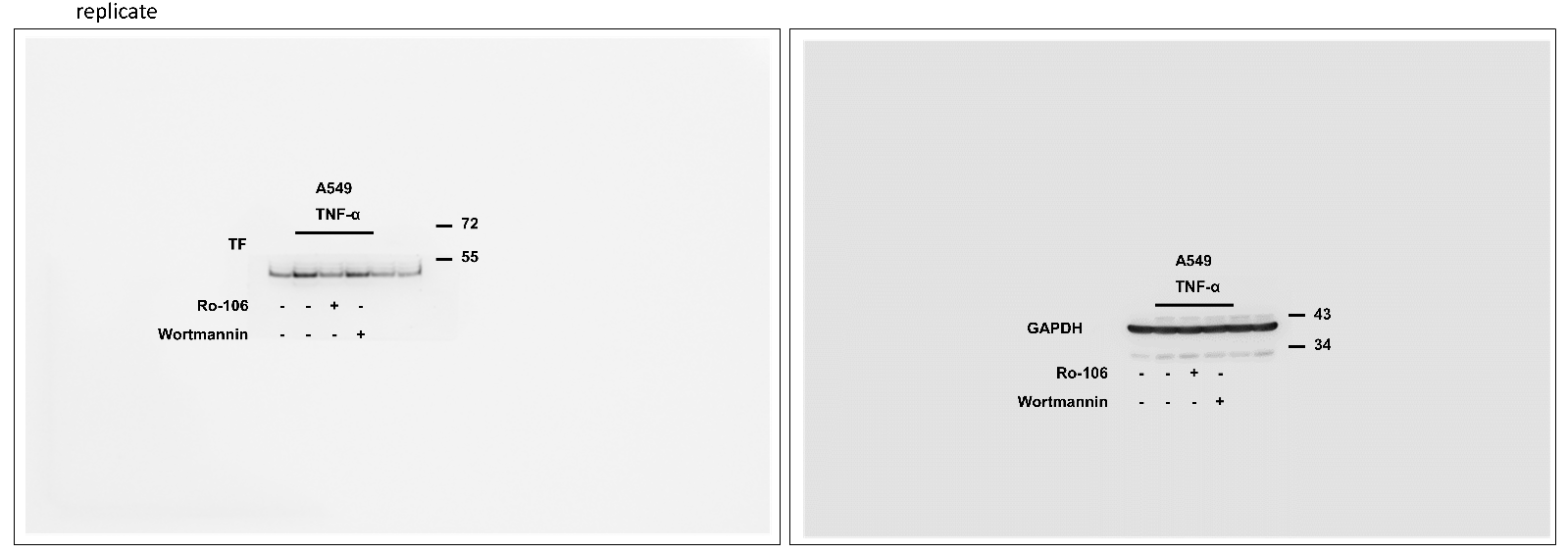


**Unedited blots and replicates of TF and GAPDH in the figure 5.** Blots of TF and GAPDH were prepared from the same nitrocellulose membrane and cut prior to hybridization with antibodies. Some lanes irrelevant to this study in the original blot were removed.


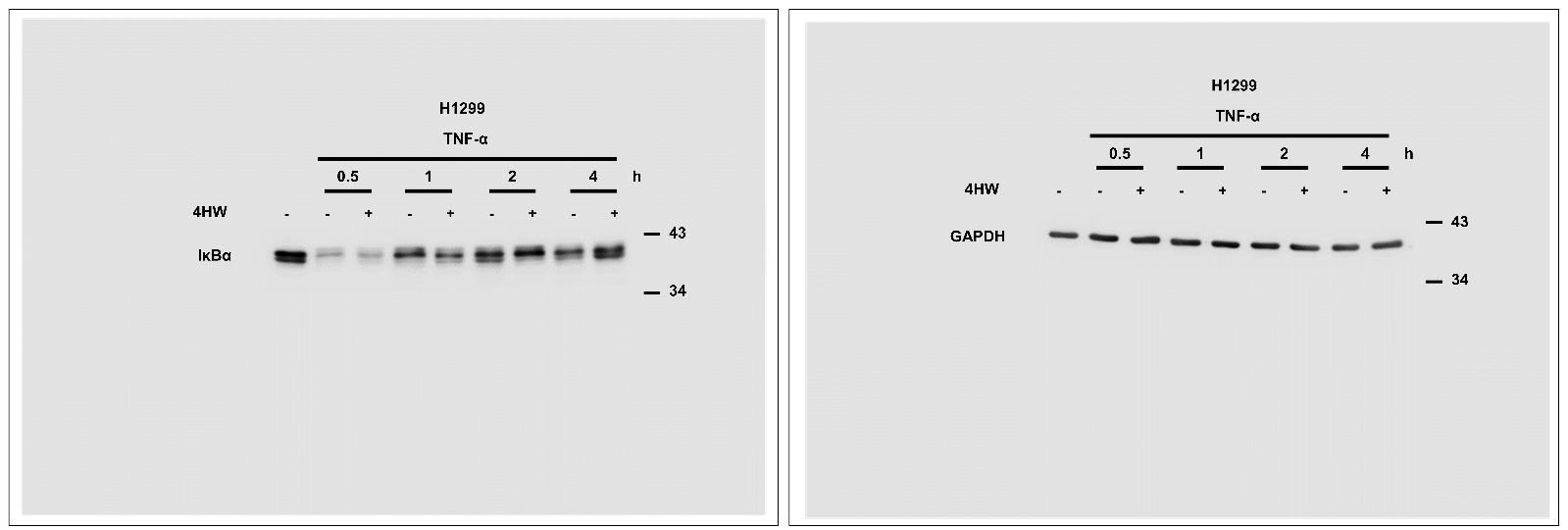

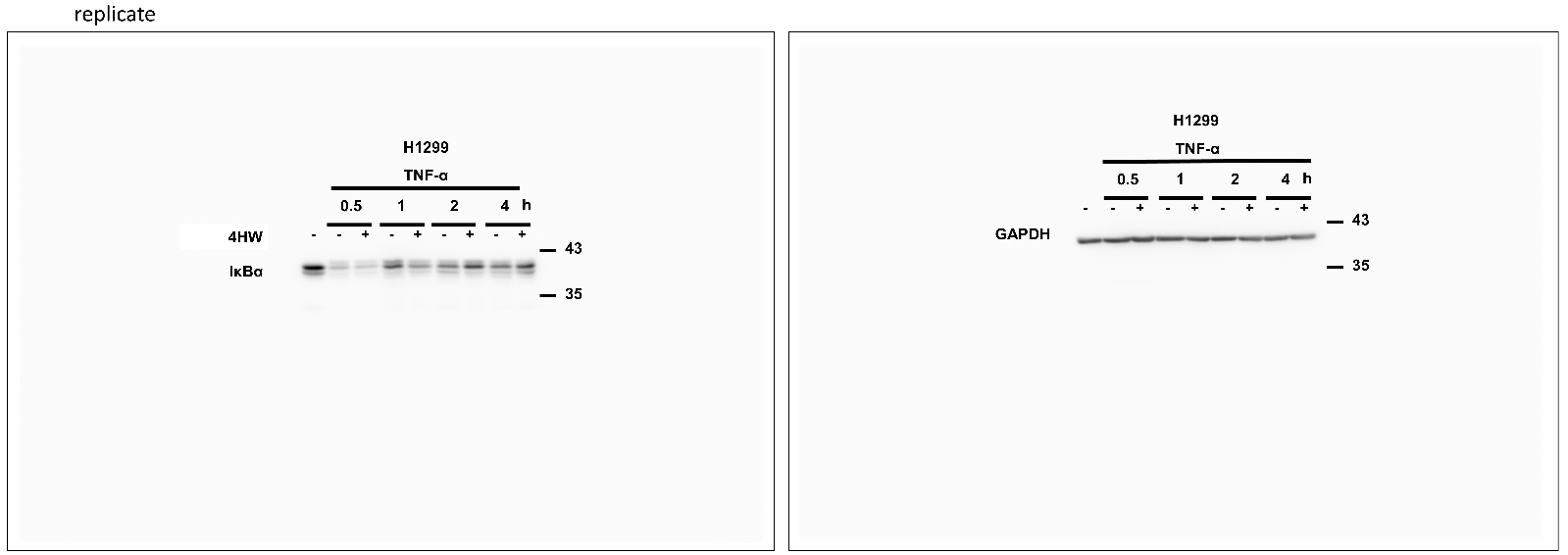

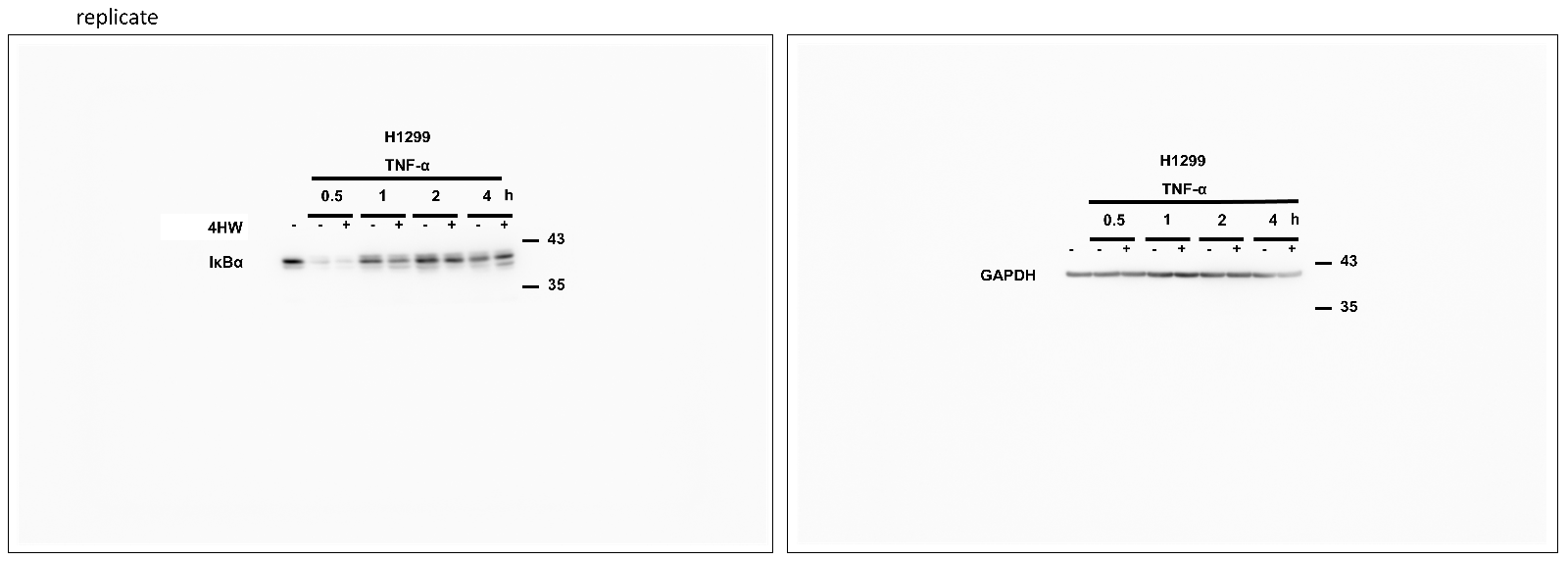

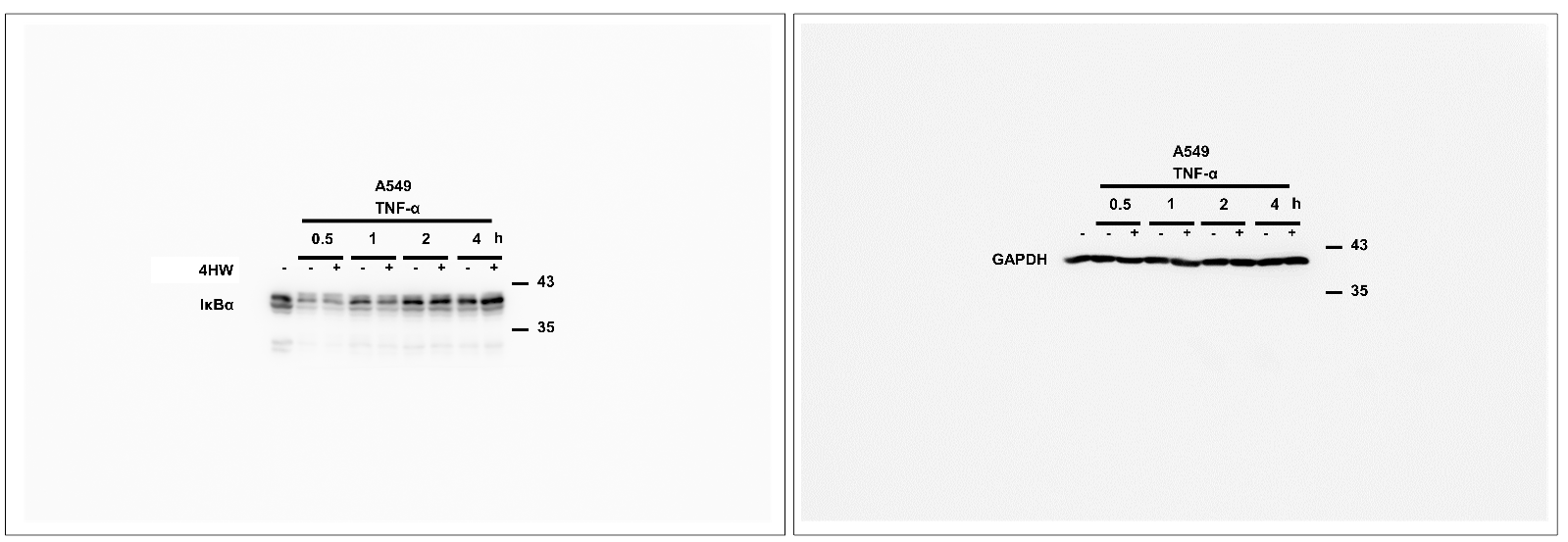

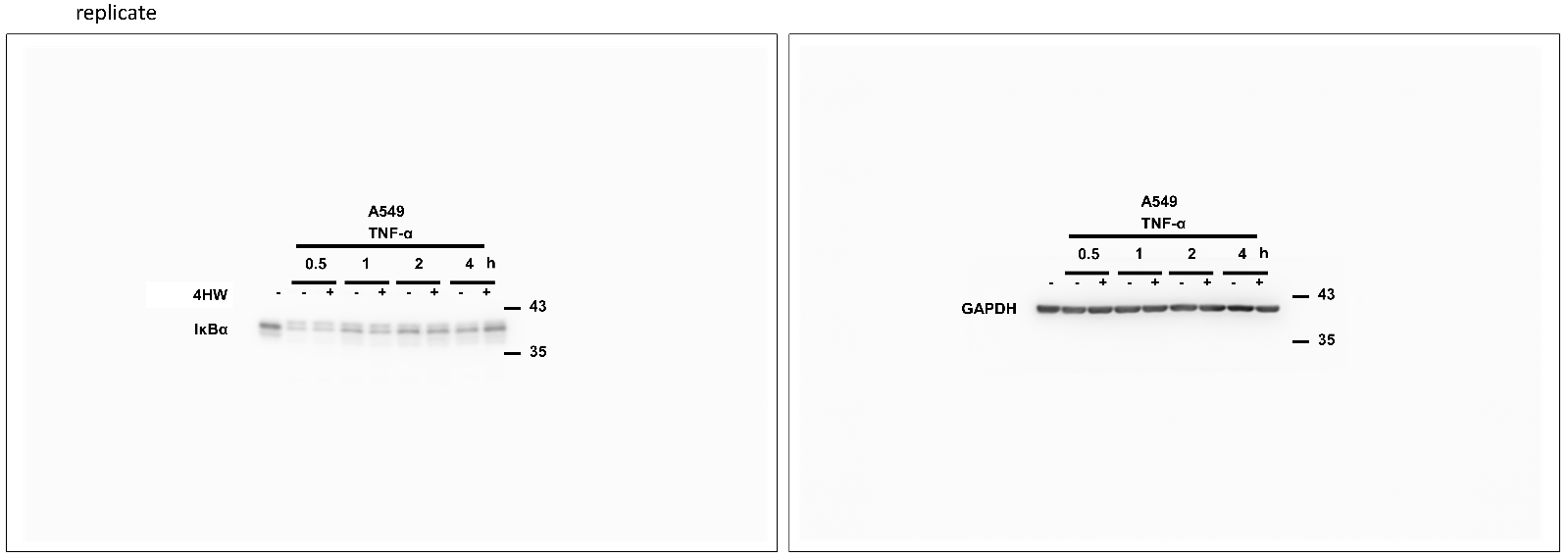

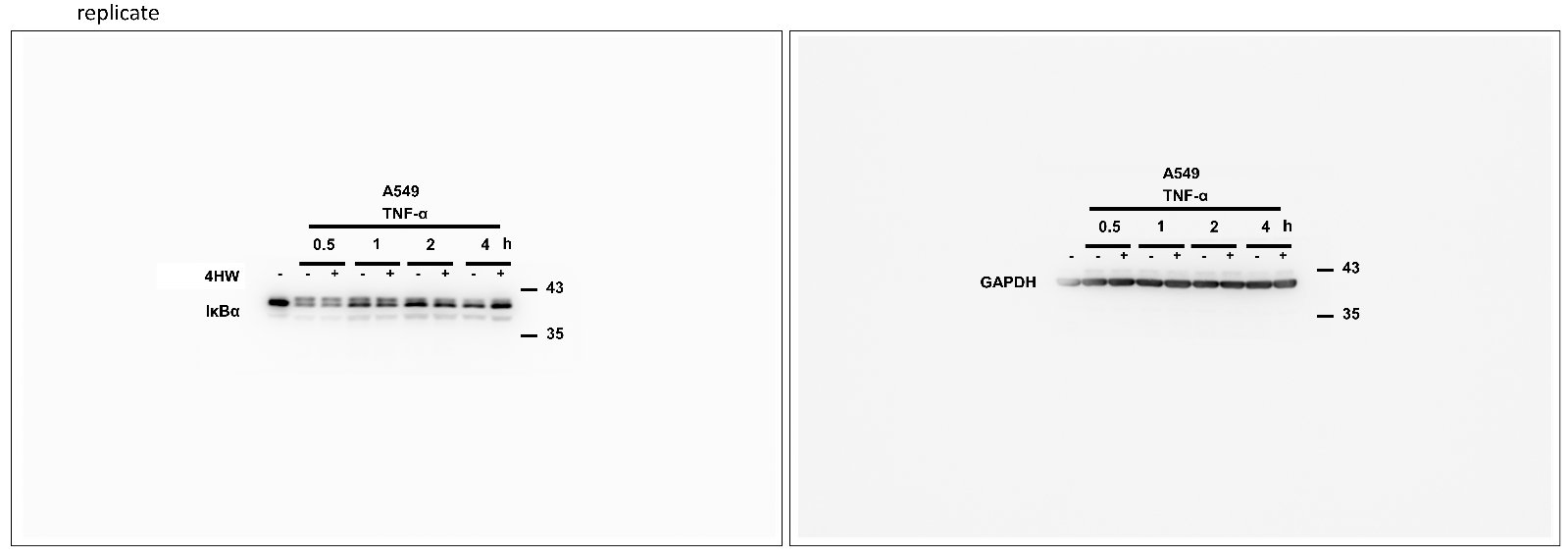


**Unedited blots and replicates of IκBα and GAPDH in figure 6.** Blots of TF and GAPDH were prepared from the same nitrocellulose membrane and cut prior to hybridization with antibodies. Some lanes irrelevant to this study in the original blot were removed.
